# Enabling Real‐Time Shape‐Sensing in Soft Robots via a Miniaturized, Single‐Signal, Color‐Tuned Soft Optical Sensor

**DOI:** 10.1002/adrr.202500124

**Published:** 2025-11-08

**Authors:** Frank Juliá Wise, Yitong Lu, Daniel Van Lewen, Riley Applebaum, Bianca Andrada, Johnathan Reamer, Sheila Russo

**Affiliations:** ^1^ Department of Mechanical Engineering Boston University Boston Massachusetts USA

**Keywords:** material robotics, optical sensing, soft robotics, soft sensing

## Abstract

Soft robotic systems require embedded sensing for closed‐loop and autonomous control; however, existing shape‐sensing approaches are limited by rigidity, bulky multisensor designs, and high computational cost. Here, we present Light‐based Ultrathin Miniature Optical Sensor (LUMOS), a miniaturized, fully soft optical sensor with a 1.25 mm cross‐section that utilizes thin film color tuning and a flexible mirror tip to deliver real‐time 3D shape information from a single red–green–blue (RGB) response. The mirror enables full signal reflection and transmission from the base, eliminating the need for onboard electronics and allowing for miniaturization. Additionally, thin film color tuning adds minimal thickness and encodes sufficient data for complete 3D shape reconstruction. Integrated into a miniaturized soft continuum robot, LUMOS achieved a predicted tip accuracy of 0.47 mm using neural network mapping. In closed‐loop operation, the robot demonstrated precise 3D targeting, maintained accuracy under external disturbances, and autonomously navigated in an in vitro lung phantom.

## Graphical abstract

A miniaturized soft optical sensor that uses thin film color tuning enables real‐time 3D shape‐sensing from a single red–green–blue (RGB) signal. When integrated into a soft robot, it enables closed‐loop control and autonomous navigation in a phantom lung environment without the need for onboard electronics, achieving sub‐millimeter accuracy through neural network‐based shape reconstruction.

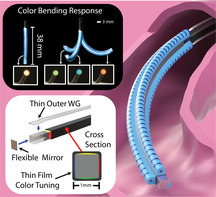

## 1 Introduction

Flexible shape‐sensing solutions that can be embedded into soft robotic platforms offer a promising way to better understand the complex, high degrees of freedom these systems exhibit [1, 2–3]. Various soft and flexible sensing architectures have been explored to provide roboticists with the real‐time feedback necessary for effective closed‐loop control [4, 5–6]. These sensor applications become even more versatile when designed for miniaturization, enabling integration into smaller platforms. This allows soft robots to navigate unstructured and hard‐to‐reach environments that rigid systems cannot access [7].

Despite these current advantages, the field of soft robotics still faces several key challenges in implementing shape‐sensing solutions. First, existing solutions utilizing fiber Bragg gratings (FBG), which employ miniaturized multiple fiber cores [8, 9–10], are made of silica glass [11] and are inherently rigid, hindering compliance and bending when embedded into soft robotic platforms. Second, fully soft solutions often rely on multiple single‐stimulus soft sensors, each designed to respond to only one type of input, such as pressure, stretch, or strain [12, 13, 14, 15–16]. Many combinations of these single‐stimulus sensors, using various sensing modalities such as capacitive, ionic, string encoding, resistive, and others [1, 17, 18, 19, 20–21], have been integrated into robot‐specific designs. However, achieving shape‐sensing this way often results in increased bulkiness and added manufacturing complexity. This limits miniaturization, as embedding multiple sensors takes up valuable space within the design, making it more challenging to adopt and implement widely. Third, the large number of signals generated by multisensor designs, combined with the complex, nonlinear responses of many sensing architectures, leads to high computational demands [10, 22]. This makes it increasingly difficult to achieve real‐time responses without relying on powerful computing systems and costly equipment.

A promising new class of soft sensors, known as soft optical sensors or soft optical waveguides (WGs), has recently been introduced [23, 24, 25, 26, 27, 28, 29–29]. These sensors present considerable promise for accurately capturing the shape of soft robotic platforms and may address many of the limitations found in current shape‐sensing technologies. They are composed of soft materials with high refractive indices (*n*), allowing light to transfer through their cores. The potential is significant, as the transmitted and received light can simultaneously encode intensity, polarization, and wavelength information within a single optical signal [30]. These sensors have been employed to track bending in robots across multiple applications by analyzing the drop in light intensity at their outputs [26, 27, 31, 32–33]. Further, their effectiveness increases significantly when paired with tuning techniques that leverage their rich informational output, enabling comparable sensing performance with fewer embedded sensors [34, 35, 36–37]. Surface roughness tuning on a waveguide has been shown to produce a bidirectional intensity response, enabling the detection of bending in two directions [38]. A similar approach using cladding that selectively blocks or transmits light has been employed to interpret twist, bending, or shear through variations in optical intensity signals [39]. These soft optical sensors can also be tuned using color pigmentation. Incorporating pigmentation into transparent optical cores enables modulation of the output signal's color in response to mechanical stimuli, such as bending or contact. Recent work has demonstrated that this approach can localize bending along 1D of the sensor by monitoring shifts in the signal's wavelength or color [40]. Furthermore, a tri‐color, internally pigmented soft optical core was able to monitor both bending and temperature differences [41]. However, a single miniaturized soft optical sensor capable of seamless integration into a soft robot for full 3D shape monitoring and closed‐loop control in a 3D workspace has yet to be realized.

In addition, machine learning models have shown great potential for managing the high informational complexity generated by multisensor, data‐dense outputs [20, 42, 43, 44–45], thus facilitating more streamlined implementation in robotic applications. Recently, neural network mapping has been employed in sensor architectures alongside optical sensors to enhance the accuracy and feasibility of decoding high‐density signal responses for improved control [41, 46, 47]. Thus, there is a promising opportunity to leverage machine learning techniques in soft optical sensing systems, utilizing the high information density provided by wavelength and color monitoring to develop a more universal shape‐sensing solution adaptable across diverse robotic platforms.

**FIGURE 1:**
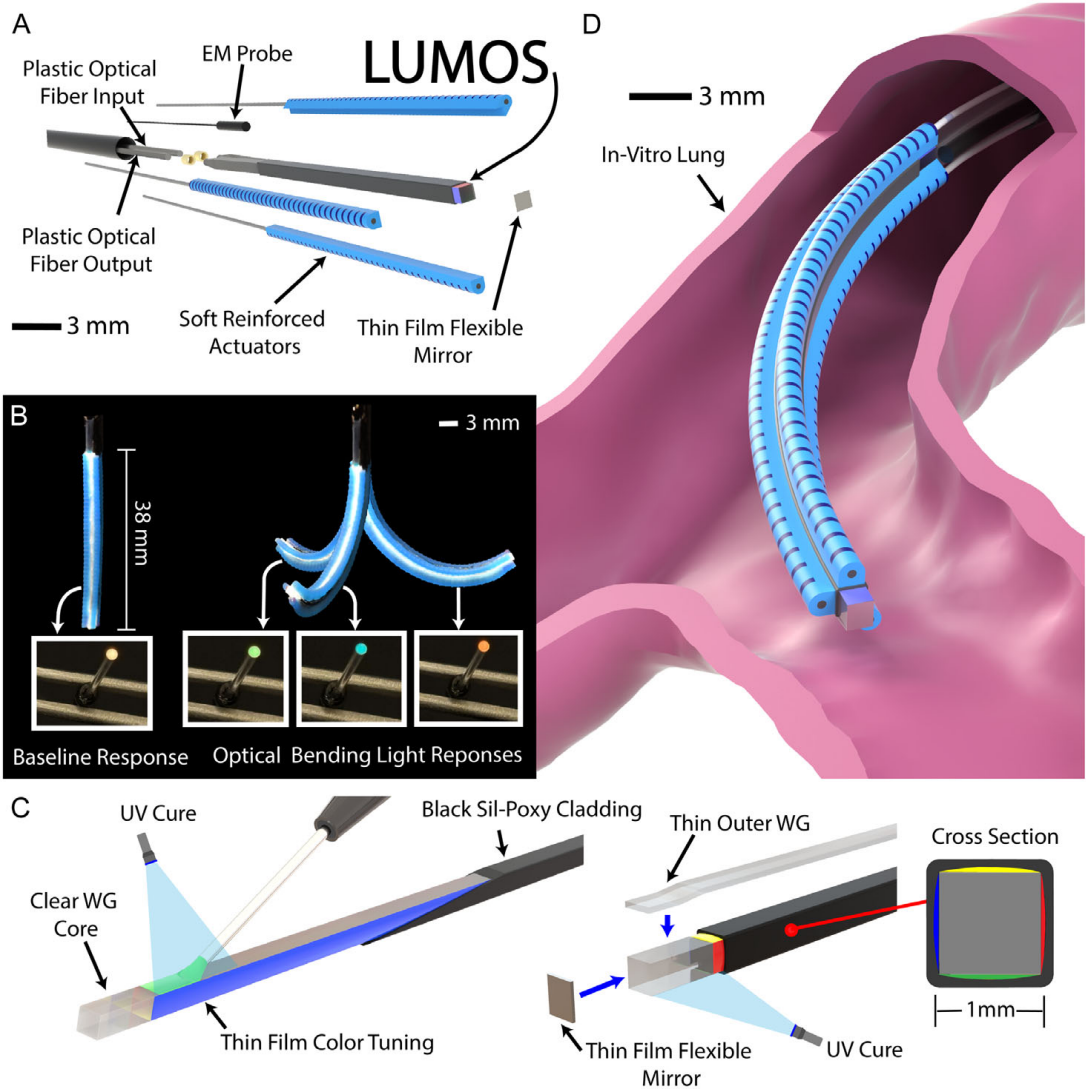
Overview of the proposed LUMOS embedded in a fully soft robot. (A) Exploded view of the soft robot showing LUMOS embedded along the neutral axis. Three soft pneumatic actuators are attached radially to enable omnidirectional bending in 3D space. A thin film mirror at the distal tip reflects the optical signal through LUMOS for sensing, which is read by the optical fiber input and output that are connected at its base. An integrated EM probe provides positional base tracking for aid in lung navigation. (B) The output color response of LUMOS when the robot is actuated in three different directions utilizing the soft pneumatic actuators. LUMOS exhibits a continuous color response with bending; representative shifts from white in the baseline straight configuration to green, blue, and red for different bending directions are shown. (C) Illustration of the thin film color‐tuning method on the clear square soft optical core and the corresponding waveguide cross‐section, showing the four deposited and cured dyed optical adhesive sides. The assembly of the distal thin film mirror and the integration of the thin outer waveguide outline the fabrication and assembly process of LUMOS. (D) Illustration of the soft robot navigating in an in vitro lung phantom environment.

In this article, we introduce a soft optical sensor that leverages thin film color tuning having a 1.25 mm cross‐section, designed for seamless integration into a miniaturized soft robot capable of full 3D shape‐sensing. We call this sensor Light‐based Ultrathin Miniature Optical Sensor (LUMOS) (Figure 1A). LUMOS contains a soft center square core that is coated with thin film layers of uniquely dyed optical adhesive (Norland Optical Adhesive NOA 65) on each of its four sides, enabling a color shift response for different bending directions and curvatures (Figure 1B). This colored film (Figure 1C) adds a minimal amount of thickness (i.e., 30 μm) to the center core's outer surface while also providing a unique color response at all points in a robot's workspace, even up to retroflexion (i.e., bending angles greater than 180°). To address the need for monitoring the transmission and reception of light from its base, a flexible thin film mirror (Figure 1C) was integrated at the tip of LUMOS. This mirror reflects light back through the square color‐tuned core, eliminating the need for large curved turns, as seen in previous designs [24, 31, 38]. We demonstrate the seamless integration of LUMOS into a 3.2 mm fully soft continuum robot (Figure 1D), which features three pneumatic actuators that enable omnidirectional bending. The robot was moved throughout its entire workspace while recording the output red–green–blue (RGB) color responses via the base of LUMOS. These responses were used to train two separate neural networks to model the robot's shape in real time. The resulting real‐time model, which achieved a predicted tip error of 0.47 mm, was then used in a closed‐loop control system to guide the robot to specific target positions in 3D space. Finally, we demonstrate the soft robot's ability to autonomously navigate to target locations within an in vitro lung environment, guided by real‐time optical color feedback.

In contrast to prior sensing approaches, these results highlight how LUMOS uniquely addresses the limitations of existing technologies. LUMOS distinguishes itself in three key ways. First, unlike rigid FBG‐based approaches that are prone to fracture and require complex, costly fabrication [48], LUMOS is entirely soft, easy to manufacture, and retains compliance during large curvatures without compromising the performance of the host robot, allowing pneumatic soft actuators to bend the embedded sensor up to retroflexion. Second, the integration of a thin film mirror at the tip of the soft sensor eliminates the need for electronics or wiring within the soft body and avoids large arched sensor paths, enabling signal transmission entirely from its base while preserving a millimeter‐scale footprint. Finally, thin film color tuning adds only minimal thickness to the sensor while generating a unique color response for all bending directions in space. This provides sufficient information to reconstruct a soft robot's 3D shape through a single soft optical channel, thereby overcoming the bulk and complexity of multisensor designs that have previously utilized soft optical WGs and other sensing methodologies. Collectively, these features establish LUMOS as a miniaturized, soft, compliant, and information‐dense sensing platform well‐suited for real‐time closed‐loop control and autonomous navigation in soft robots.

## 2 Results

### 2.1 Operating Principle and Experimental Implementation of the LUMOS Thin Film Color‐Tuned Core

Soft optical WGs are characterized by their ability to transfer light through their core, a function enabled by the refractive index contrast between the core and the surrounding cladding material. The Snell's law governs the behavior of light at the interface between the core and cladding of a WG, using their respective refractive indices (*n*_1_ for the core and *n*_2_ for the cladding) to determine the critical angle (*θ*_c_) required for total internal reflection, enabling light propagation within the core, where $\theta_{c}=\arcsin\;\left(\frac{n_{2}}{n_{1}}\right)$. When light travels through a WG core, it interacts with the cladding at an incident angle *θ*_*i*_. If the incident angle *θ*_*i*_ exceeds the critical angle *θ*_c_, the light remains confined within the core and propagates through it. Conversely, if *θ*_*i*_ is less than *θ*_c_, the light escapes and diffuses into the cladding. The larger the difference in the two refractive indices, the larger the curvatures a WG can experience before losing its intensity.

**FIGURE 2:**
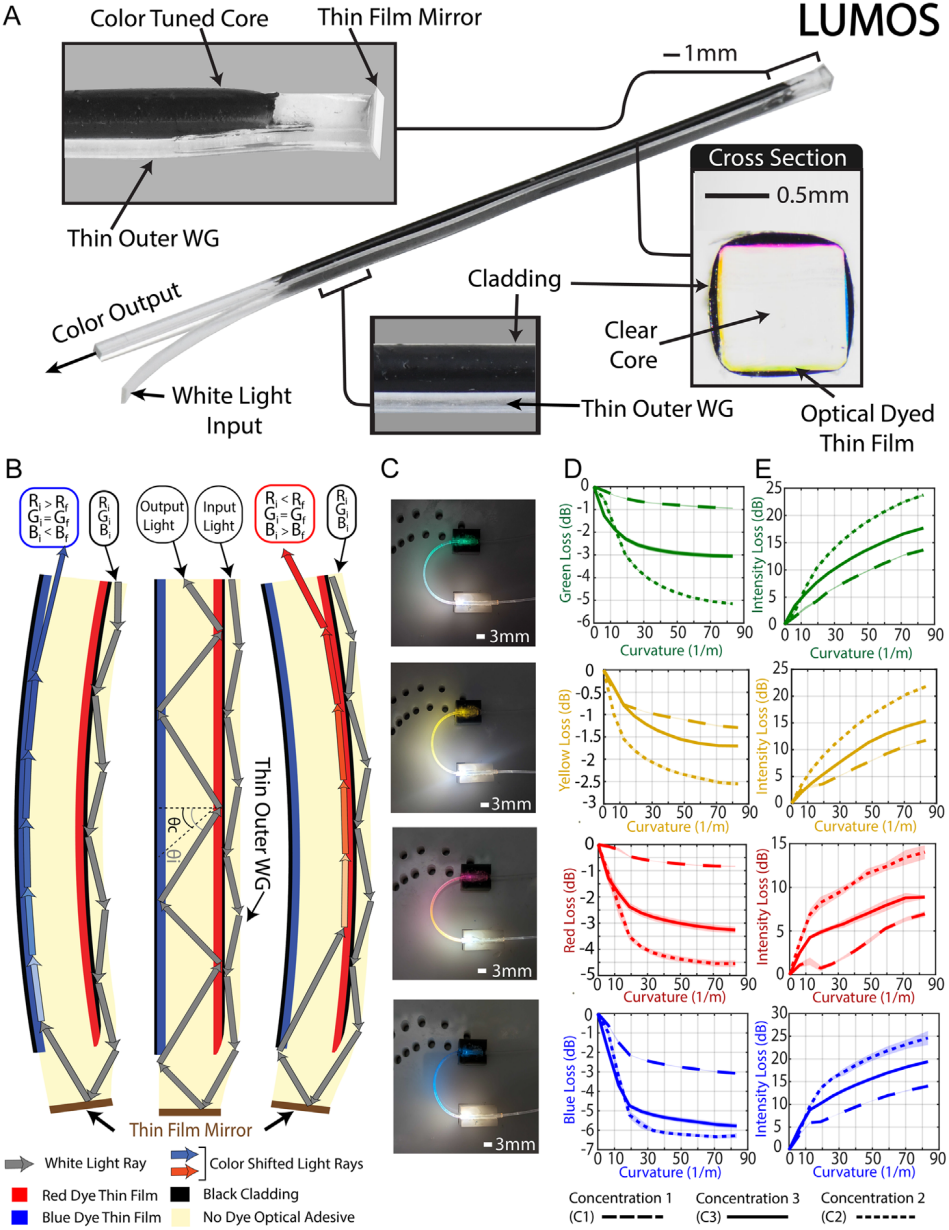
LUMOS design, ray propagation diagram, and dye concentration thin film plots. (A) Design of LUMOS, including the magnified view of the tip of the sensor showing the connection point between the thin outer waveguide and the color‐tuned core in relation to the thin film mirror. A magnified image of the cross‐section of the central color‐tuned core is shown, encapsulated by the outer black cladding. The white‐light input location and the corresponding color output are also indicated. (B) The physics ray diagram is shown. The light enters the thin outer waveguide, reflects off the thin film mirror, and back through the color‐tuned core. The sensor is depicted in three configurations, straight, redshifted bending, and blueshifted bending, illustrating how deformation concentrates the rays on the side opposite the bend, resulting in a color shift due to the thin film color‐tuned layers. (C) Color dye thin film characterization testing setup with each thin film dye core bending to retroflexion. (D) Plots showing the predominant color loss with increasing curvature for four thin film colors, each tested at three concentration levels (C1–C3). The plots are color‐coded according to their respective dye colors and concentrations distinguished by unique dashed and nondashed lines. (E) Plots showing the intensity signal loss with increasing curvature for four thin film colors, each tested at three concentration levels (C1–C3). The plots are color‐coded according to their respective dye colors and concentrations distinguished by unique dashed and nondashed lines.

LUMOS features a clear WG core (Figure 2A) made of NOA 65, chosen because of its high refractive index of 1.56, which is considerably higher than its black silicone adhesive cladding (Sil‐Poxy), with a refractive index of ≈1.44, measured using a Woollam VASE Ellipsometer for the visible spectrum range. Surrounding the clear core are four uniquely dyed (red, green, yellow, and blue) soft NOA 65 thin films, shown in Figure 2A cross‐section. As an initial step in the fabrication process of these films, a colored dye is added to the uncured liquid NOA 65 and mixed to uniformly suspend the dye in the material. The incorporation of pigmented dye into the optical material transforms it from transparent to one that selectively alters the color of transmitted white light that passes through it. This occurs as the suspended pigment absorbs specific wavelengths while allowing others to pass through, thereby modifying the spectral composition of the transmitted light. This dyed adhesive is then deposited on the surface of the square clear core using a syringe (Figure 1C) and is able to maintain its position on the surface due to the surface tension of the liquid. The uncured dyed liquid NOA 65 is then cured using ultraviolet (UV) light, resulting in its full bond to the clear core, leaving a 30 μm thin film on its surface (Figure 1C). By repeating this on each side of the core using a different color‐induced NOA 65 (Figure 1C), it results in a structure we call the color‐tuned core (Figure 2A, cross‐section).

When the color‐tuned core bends, the input white light concentrates on the opposite side of the direction of bending [49]. This results in a larger concentration of light passing through the thin pigmented film rather than the center clear core. This phenomenon is illustrated in a 2D ray propagation diagram (Figure 2B), in which three scenarios are shown. In Figure 2B Left, the color‐tuned core bends with the blue thin film opposite the side of bending. This changes the incoming white light (*R*_*i*_,*G*_*i*_,*B*_*i*_) to shift in color to blue, increasing the output *B*_f_ signal. In Figure 2B Right, the color‐tuned core bends in the opposite direction, resulting in a larger concentration of light propagating through the red thin film layer, resulting in a white‐to‐red color shift, increasing the *R*_f_ output signal. When the color‐tuned core is in its straight configuration, the thin films minimally affect the signal response, resulting in no color shift from the input to the output (*R*_*i*_,*G*_*i*_,*B*_*i*_) = (*R*_f_,*G*_f_,*B*_f_) (Figure 2B Middle). When the color‐tuned core is bent between these thin film directions, the concentration of light is shared among multiple films, leading to color mixing, as shown in Supporting Movie S1, where only the color‐tuned core is shown being bent in 3D space with the resulting color light output shown. This color mixing facilitates a unique signal for any direction in which the sensor is bent in 3D space.

Adding excessive dye to the thin films results in significant light absorption, thus reducing the strength of the propagated light during bending and impacting the ability to generate a reliable signal in LUMOS. To define the specific concentration of dye that yields both a high‐resolution color shift when bent and a limited amount of light loss, three concentrations (C1‐C3) for each colored dye were tested (Supporting Table S1). Twelve clear cores (three for each color) were fabricated, each incorporating a single thin film induced with a specific dye concentration. The square single thin film color‐tuned cores were bent in constant curvature up to retroflexion (Figure 2C), with the thin films positioned on the opposite side of the bending. The resulting RGB values and signal intensity were recorded using an Adafruit TCS34725 color sensor, and the curvature values were predetermined based on the specifications of the testing setup design (Figure S1). The predominant RGB value was monitored for each specific dye color tested (i.e., Red (R) for red dye, Blue (B) for blue dye, and Green (G) for green dye) and the mean value between Red and Green for yellow dye (Y). To quantify changes in both overall light intensity and the RGB signal during bending, we employed the loss function $L=10\log\;\left(\frac{I_{b}}{I_{c}}\right)$, where *I*_b_ is the baseline intensity measured at the core's straight, no‐curvature configuration and *I*_c_ is the recorded intensity under bending. This decibel‐based formulation, commonly used to describe optical attenuation, was previously employed in our earlier work [36, 38, 50] and is applied here to both the predominant RGB channel values and the overall light intensity, which were plotted as a function of curvature (Figure 2D, E). The resolution of each thin film concentration is defined as the mean of the rate of change in optical loss of the dominant RGB signal, with respect to curvature, expressed as $\text{Resolution}=\text{mean}\left(\frac{d(\text{loss})}{d(\text{curvature})}\right)$. This definition reflects the average slope of the loss to curvature relationship. A higher value indicates that a given increment in curvature results in a proportionally larger change in optical loss, thereby denoting a finer sensitivity to curvature variations. By employing the mean slope as a metric, this approach enables consistent and quantitative comparison of different dye concentrations in terms of their ability to discern curvature into measurable optical signals. The resolution of each concentration with respect to curvature is shown in Supporting Table S2. The predominant color loss measurements (Figure 2D) indicate that the higher the concentration of dye, the larger the shift in predominant color response, resulting in a higher resolution with respect to curvature. Conversely, the intensity plots (Figure 2E) inform us that the larger the concentration of dye, the larger the loss is in the overall signal intensity with regard to the corresponding curvature. This is inversely true with a low concentration of dye, in which the predominant color shift in relation to curvature is limited, yet keeps a larger light intensity for larger curvatures. This is consistent across all concentrations and dyes.

To quantify the variability in the signal response due to excessive light intensity loss at larger curvatures, an additional test was conducted. Each of the 12 sensors with specific dyes and concentrations was bent to a maximum bending curvature of 83.33 m^−1^, and the RGB response was recorded. This was repeated 15 times with each sensor to monitor the variability in the response. The signal‐to‐noise ratio (SNR) of these responses was then calculated to evaluate the signal's reliability. The signal is defined as the mean change in transmitted intensity relative to the baseline straight configuration (*μ*_signal_), while the noise is defined as the standard deviation of these repeated measurements (*σ*_noise_) at 83.33 m^−1^. The SNR was then calculated as: $\text{SNR}=\frac{\mu_{\text{signal}}}{\sigma_{\text{noise}}}$. This definition presents the relationship between the amount of signal change for the RGB response in relation to the overall noise of the signal. A higher value for SNR indicates less disturbance of the overall signal change due to noise when a lower value indicates the noise is having a larger effect on the overall signal response. The results for these calculations are given in Table S3. From these results, we see that C3 SNR values are consistently the lowest, indicating that the large intensity loss experienced is causing variability in the signal, while C2 and C1 show higher SNR values and thus more consistent responses. For LUMOS's color‐tuned core, a balanced concentration, C2, was chosen for all thin films, as it provided a significant color shift up to retroflexion, unlike C1, while maintaining a stronger overall signal intensity and SNR value than that of the highest concentration, C3.

### 2.2 Fabrication and Assembly of the Thin Outer Waveguide and Flexible Thin Film Mirror

Previous soft WG designs have either employed large, arched curvatures to send and retrieve a signal from outside the robot's environment or embedded rigid electronics, such as LEDs, photodiodes, and photoreceivers, at both ends of the sensor [24, 25, 40, 41, 51, 52]. The incorporation of a flexible thin film mirror at the end of a soft optical sensor presents an effective way to eliminate the need for electronics, external wiring, or arc turns that can limit miniaturization and increase implementation complexity.

To realize this concept, LUMOS's design, shown in Figure 2A, consists of three main components: a center color‐tuned core, a thin outer WG, and a flexible thin film mirror. The first step in the assembly of these three parts is to coat the color‐tuned core with dyed black Sil‐Poxy as shown in Figure 1C. To facilitate deposition, the black Sil‐Poxy is mixed with a solvent in a 1:2 ratio to reduce its viscosity, allowing for a 0.1 mm coating around the color‐tuned core (Figure 2A cross‐section). This black cladding serves as a barrier between the outer thin WG and the center color‐tuned core, limiting any cross‐talk of light that could potentially transfer between the two. The tip of the color‐tuned core is deliberately left uncoated in cladding material, leaving it exposed for connection.

The thin outer WG, with a thickness of 0.15 mm, carries the input white light to the end of the sensor and is bonded to the outside of the black cladding using Sil‐Poxy, thus securing it to the outer wall of the color‐tuned core. This thin WG is then additionally bonded to the tip of the sensor on the exposed section of the color‐tuned core (Figure 1C) using NOA 65. This bonding allows incoming white light to be transferred through the thin outer waveguide into the color‐tuned core only at the sensor's end. The mirror, a flexible thin sheet of polymer reflective film (3M ESR Reflector Film), is cut to shape using a CO_2_ laser and bonded to the tip of the sensor using additional UV curable NOA 65 (Figure 1C), shown fully connected in the magnified tip view (Figure 2A). The integration of this mirror marks the completion of LUMOS, and its fabrication and assembly are demonstrated in Supporting Movie S2. The ray propagation diagram (Figure 2B) illustrates the functionality of this mirror; the input white light that propagates on the outside WG of the black cladding is able to reach the tip of the sensor and diffuse into the color‐tuned core at the exposed WG section. It is then reflected back through the color‐tuned core by the thin film mirror, where it is affected by the thin film color tuning, and monitored at its base. This solution yields a design capable of sending and receiving signals from outside a robot's workspace environment while maintaining a maximum cross‐sectional dimension of just 1.25 mm.

### 2.3 Robot Design, LUMOS Integration, and Signal Evaluation

**FIGURE 3:**
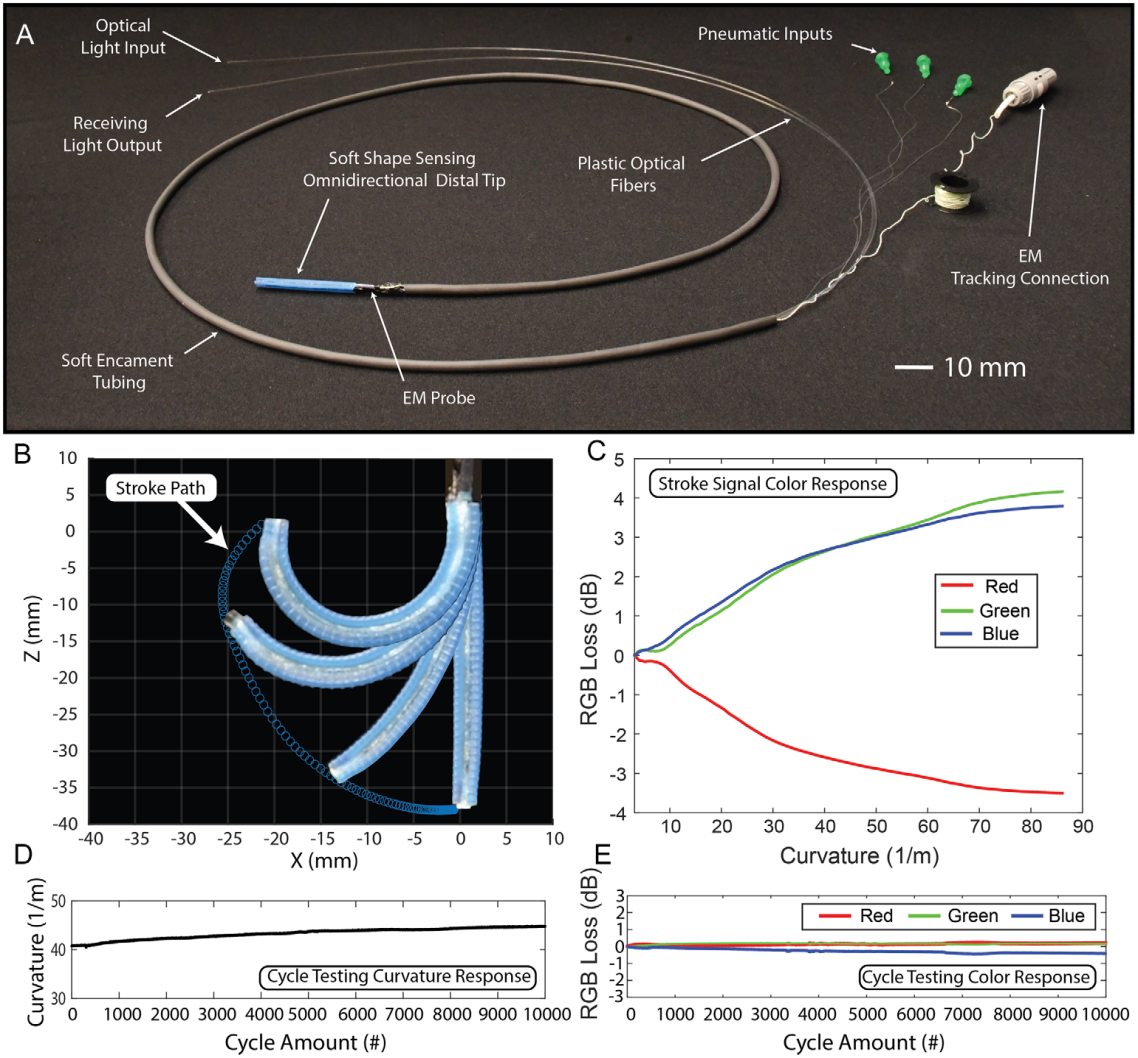
Robot design, LUMOS integration, and signal evaluation. (A) The photographed robot shows the soft distal tip with EM probe embedded at its base, soft encasement tubing, optical fibers for input and output of light signals, and pneumatic and EM probe connections all located away from the distal tip. (B) Stroke plot of the robot being actuated utilizing a single soft pneumatic actuator up to retroflexion with the robot superimposed. (C) Plot of the loss of each RGB value with respect to curvature for the stroke test up to retroflexion. (D) Cyclic testing results showing the change in curvature of the robot over the ten thousand actuation cycles. (E) The resulting change in the RGB signal over the ten thousand cycles represented by the loss with respect to the initial actuated RGB response.

The main body of our soft robot consists of a soft distal tip, followed by a 2 m passive segment (Figure 3A). The soft distal tip, 38 mm in length, consists of three soft reinforced pneumatic actuators bonded radially around LUMOS, keeping it at the robot's neutral axis, resulting in a cross‐sectional diameter of the robot of 3.2 mm. The combined pressurization of these actuators results in the desired omnidirectional bending of the robotic tip, while LUMOS facilitates the real‐time color‐shifting response when bending (Figure 1B) (Supporting Movie S3). The passive segment of the robot consists of soft encasement tubing, through which two optical fibers are fed to send and receive the light response from LUMOS's base to be monitored outside the robot's workspace, in addition to holding the pneumatic tubing and electromagnetic (EM) probe connection (Figure 3A).

To assess the functionality of LUMOS in a soft robotic environment, we conducted two targeted experimental evaluations. The first aimed to monitor the RGB response as the robot is bent to the point of retroflexion, a critical condition for evaluating signal behavior that might be experienced in a soft continuum robot platform. A single actuator on the robot was pressurized to 300 kPa, which enabled it to reach retroflexion, as shown in the stroke plot with the robot superimposed in Figure 3B. The RGB signal response was monitored during this actuation using an LED (XLamp CXA2520 White) at the fiber's input and the Adafruit color sensor at the output of the fiber. The loss of the three RGB values was individually calculated, using the straight initial position as the baseline signal, and shown in Figure 3C in comparison to the robot's curvature. From this plot, we can see the monotonic signal response for all colors, having a steady increase or decrease in signal up to retroflexion, demonstrating the ability to use the RGB color response to accurately map the robot's bending, even at large curvatures.

The second test evaluated LUMOS's signal reproducibility over a high number of bending cycles, aiming to assess its reliability and stability under repeated deformation. The robot was actuated 10,000 times to a pressure of 220 kPa, resulting in a starting curvature of 40.5 m^−1^ and finishing at a curvature of 44.9 m^−1^ (Figure 3D). The RGB values were recorded for each cycle, with the first data point at a maximum curvature of 40.5 m^−1^ serving as the baseline for the calculated loss during testing. The loss remained consistent throughout the entire cycling process, with deviations of 0.2 dB in red, 0.2 dB in green, and 0.4 dB in blue, as shown in Figure 3E. This consistency demonstrates the repeatability of the RGB color response, even after 10,000 cycles. This test confirms the robustness and stability of LUMOS over extended use when embedded into a soft robotic platform.

### 2.4 Shape‐Sensing Characterization and Validation

**FIGURE 4:**
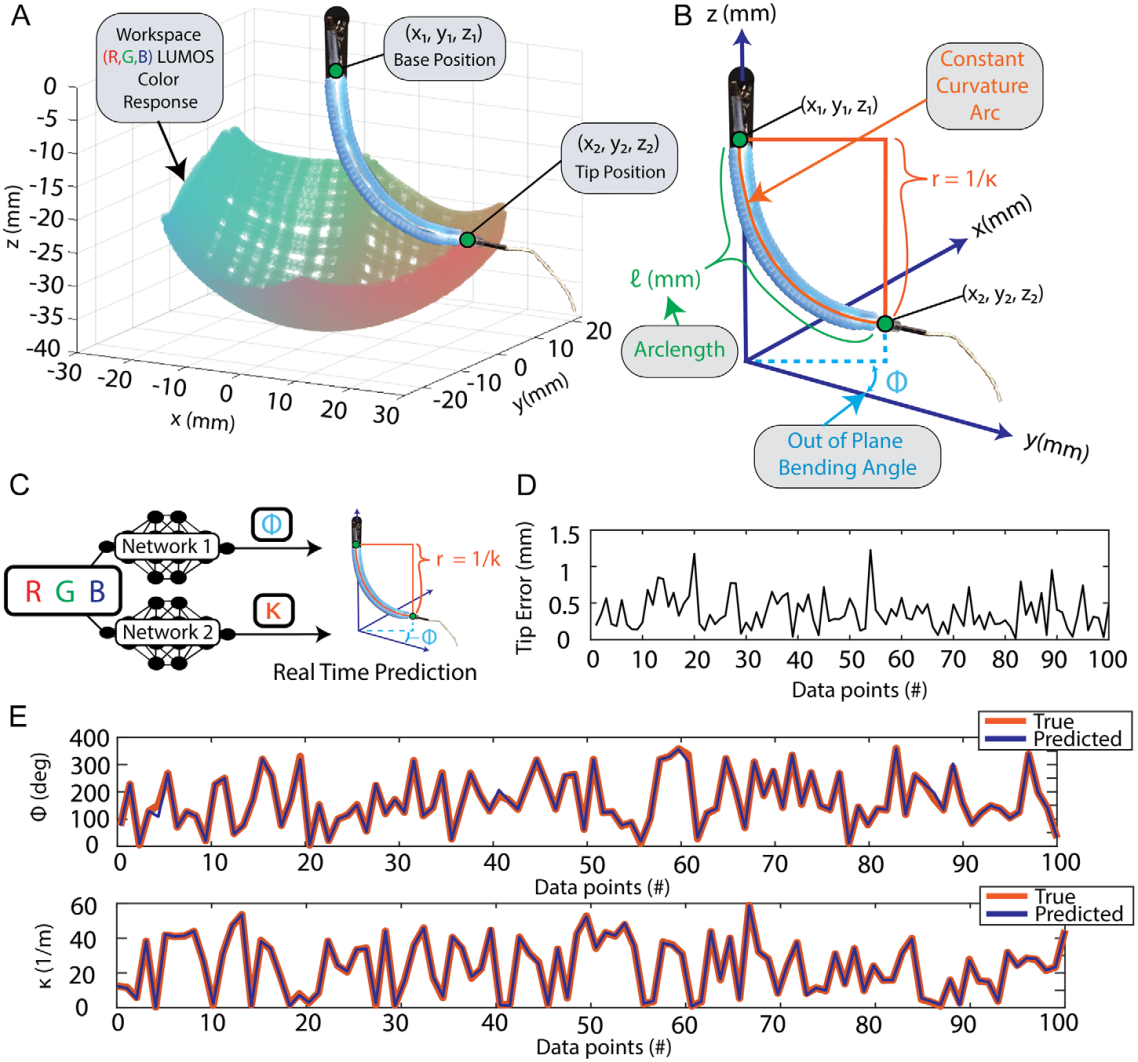
Calibration and neural network validation. (A) Calibration of the robot's shape in 3D space is performed by sequentially actuating the pneumatic chambers to move the robot throughout its entire workspace while collecting both the base and tip position data. Each recorded position corresponds to a distinct LUMOS sensor color response, illustrated by the color of the points in the workspace plot. (B) The robot with the superimposed constant curvature parameters is shown. The tip and base positions are used to define the parameters, including the bending angle Φ and curvature *k*, which together describe the robot's shape. (C) Illustrated neural network diagram showing the training process, where separate networks are used to predict the shape parameters Φ and *k*. Each network is trained on the corresponding RGB values for specific constant curvature parameters. (D) Error values between the true and predicted robot shape of the tip location of the robot for 100 unseen data points. (E) Validation result of the proposed neural networks true vs. predicted Φ and *k* values for 100 unseen data points.

To calibrate the color‐shifting response of LUMOS, we used the soft robotic platform as a controlled testbed. The three soft reinforced actuators were individually pressurized to bend the robot in 3D space using pneumatic regulators (ITV0030‐2N, Compact Electro‐Pneumatic Regulator) to control their internal pressure. To characterize the shape of the robot, two EM tracking probes, one at the base and one at the tip of the robot, are used to obtain the robot's base (*x*_1_, *y*_1_, *z*_1_) and tip position (*x*_2_, *y*_2_, *z*_2_), as shown in Figure 4A. The robot was designed to bend in constant curvature [53, 54], defined by a singular arc that runs through its neutral axis (Figure 4B). The constant curvature values Φ and *k* that define a constant curvature arc in 3D space were then calculated using Equation (1) and (2), assuming the arc length (*l*) is constant at 38 mm. These physical parameters are superimposed on the actuated robot and are also shown in Figure 4B.

(1) $$\kappa =\frac{2\sqrt{{\left(x_{2}-x_{1}\right)}^{2}+{\left(y_{2}-y_{1}\right)}^{2}}}{{\left(x_{2}-x_{1}\right)}^{2}+{\left(y_{2}-y_{1}\right)}^{2}+{\left(z_{2}-z_{1}\right)}^{2}}$$

(2) $$\Phi =\arctan\;2\left(\frac{y_{2}-y_{1}}{x_{2}-x_{1}}\right)$$

The robot was cycled through two preprogrammed combinations of pressures, utilizing all three actuators to enable the robot to move freely through the workspace. During both cycles, only two of the three actuators were pressurized simultaneously, while the third remained unpressurized until the next actuation combination pair occurred. In each cycle, one actuator moves through its full range of motion while the second actuator steps incrementally through its range. This process is repeated for all possible pairs of actuators, ensuring that every combination is tested. The first preprogrammed combination had defined larger ranges of motion with respect to curvature, resulting in the robot reaching a max curvature of 55 m^−1^ and a full range of Φ values from 0° to 360°. Data was continuously collected throughout the cycling process, resulting in 22,000 data points, each containing the RGB values corresponding to the respective Φ and *k* values reached by the robot. The second cycle collected 10,000 data points for smaller curvatures with a max curvature of *k* equal to 11 m^−1^, still accomplishing the full range of Φ values from 0° to 360°, ensuring there is a large amount of data for both the larger and smaller curvatures in the workspace for the full Φ range. The resulting workspace and signal response plot is shown in Figure 4A, where each point represents the LUMOS's color output, visualized through its corresponding RGB color. This illustrates the designed thin film color‐shifting response as it transitions across different positions within the workspace confirming the increase in color intensity as it shifts away from white light at larger *k* values, along with a distinct color change across varying Φ values, resulting in a unique optical response for each point within the robot's workspace.

A neural network mapping approach was selected to predict Φ and *k* values, given the robot's ability to generate a large and consistent dataset. This training was conducted using the MATLAB Deep Learning Toolbox (MathWorks Inc., 2024). The model design and implementation are described in the Materials and Methods section (Neural Network Implementation). These neural networks, illustrated in Figure 4C, allow for a real‐time prediction of the shape of the device by having an RGB value as input and a *k* and Φ value as output, only taking 0.024 s to run the data through both networks. The prediction accuracy of these networks is confirmed by using (10%) of the total dataset as unseen data points within the robot's workspace, a hundred points of which are shown in (Figure 4E). The average mean error between the true and predicted values is 0.39 m^−1^ for the *k* prediction and 4.8° for the Φ prediction.

The predicted tip position of the robot using the predicted Φ and *k*, and arc length *l* values was determined using the constant curvature forward kinematics given in Equation (3) :

(3) $$(x,y,z)=\left(\frac{\cos\;(\Phi )(1-\cos\;kl)}{k},\frac{\sin\;(\Phi )(1-\cos\;kl)}{k},\frac{\sin\;kl}{k}\right)$$

The average tip error between the true and predicted values was then calculated and is given for 100 predicted points shown in Figure 4D. The resulting mean total tip error across all unseen data points was 0.47 mm, having a 95th percentile error of 0.91 mm, and a standard deviation of 0.32 mm, showing the robustness of our dual network approach. A video demonstrating the robot's real‐time 3D shape‐sensing response both from robot actuation and external manual disturbances in relation to its true shape, utilizing these two neural networks, is provided in Supporting Movie S4.

### 2.5 Closed‐Loop Control

**FIGURE 5:**
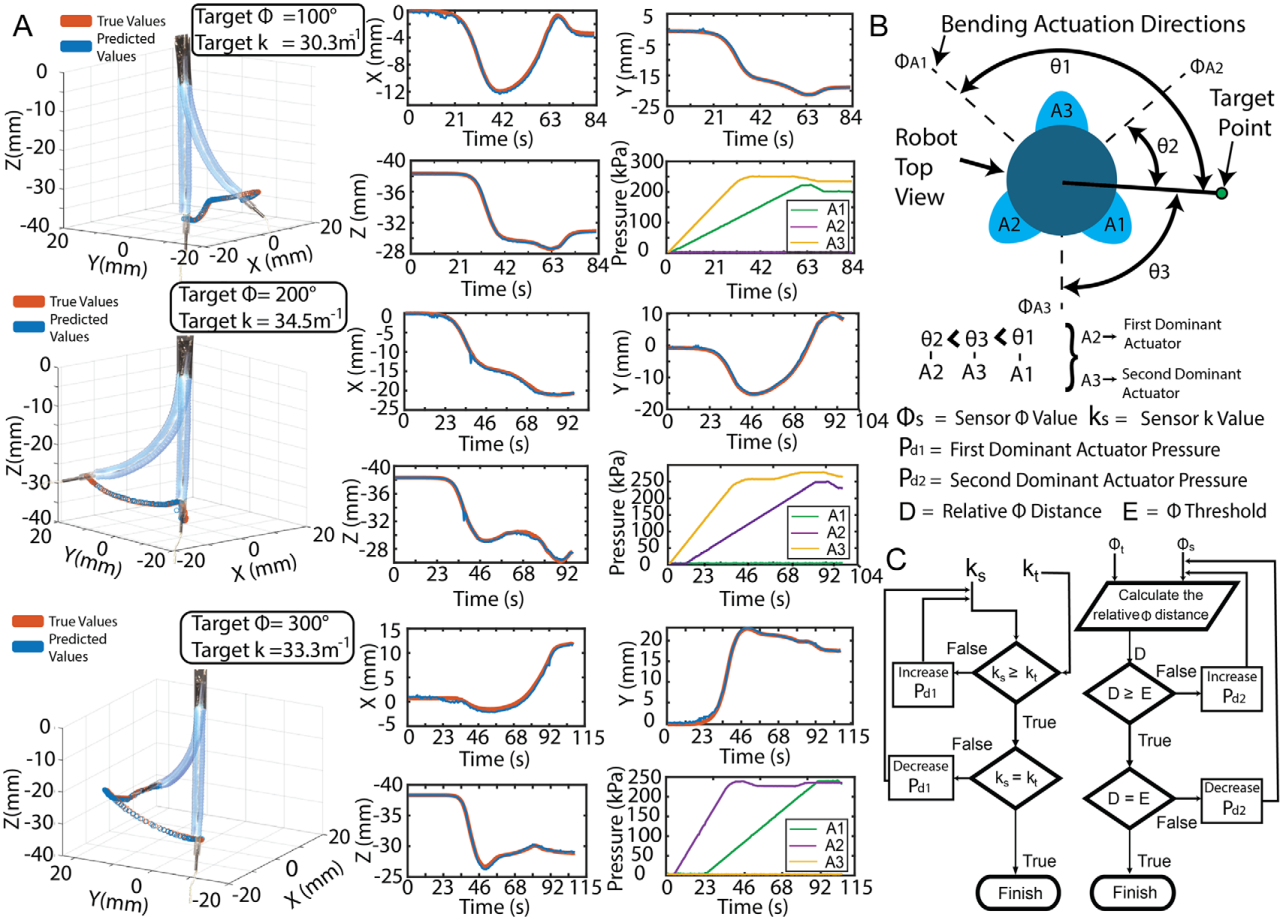
Closed‐loop control free space point following. (A) The true and predicted tip position of the robot during its closed‐loop navigation to three different target locations in space, with the robot superimposed, are shown on the left. The *X*, *Y*, and *Z* plots of the true and predicted values of the tip of the robot for each navigated point are also shown with respect to time (Right). Additionally, the resulting internal pressure of the three pneumatic actuators (A1, A2, and A3) during the navigation with respect to time is shown (Right). (B) Illustration of the top view of the robot showing the three actuators (A1–A3) and their respective bending directions when pressurized. The angular distance (Φ) from each actuator direction to the target point, denoted as *θ*_1_, *θ*_2_, and *θ*_3_, is indicated. These values are used to identify the first and second dominant actuators, dependent on their relative angular distance to the target point, enabling the robot to reach its desired target. (C) The independent closed‐loop control block diagram for Φ and *k* values of the robot to reach the target $\Phi_{t}$ and *k*_t_ values to navigate to a desired point in 3D space. The predicted values ($\Phi_{s}$, *k*_s_) are compared against the target values ($\Phi_{t}$, *k*_t_) to guide the robot toward a desired point. For curvature *k*, target and predicted values are directly compared, while for bending angle Φ, the relative distance between $\Phi_{s}$ and $\Phi_{t}$ is evaluated against a predefined threshold to determine whether actuator pressure should be increased or decreased. The two dominant actuators are adjusted independently following this control structure until both $\Phi_{s}$ and *k*_s_ converge to their respective targets.

Hereafter, we demonstrate the capability of LUMOS to guide the soft robot to target locations in 3D space using the shape‐sensing response in closed‐loop control (Figure 5A). A constant curvature arc is defined from the base of the robot to a desired point location in the robot's workspace, calculated using Equation (1) and (2). This results in a target curvature value *k*_*t*_ and a Φ value $\Phi_{t}$ for our robot to attempt to reach. All three actuators contribute to the robot's omnidirectional movement; however, only two actuators at a time are pressurized to reach a target arc. The two selected actuators are referred to as the first and second dominant actuators. The first dominant actuator is selected as the one which, when actuated alone, brings the robot tip closest to the desired position, having the lowest relative Φ distance to the target $\Phi_{t}$ (Figure 5B). As a result, it primarily governs the curvature *k* of the robot, since its actuation direction aligns most closely to the curvature when actuated. The second dominant actuator, in contrast, has less influence on the tip's distance to the point but plays a more significant role in adjusting the robot's Φ. This is due to the fact that its direction of influence is not aligned with the target position and, thus, is more effective at rotating the tip to the desired $\Phi_{t}$ value and thus is the control for Φ.

The control loops for both the first dominant and second dominant actuators are run independently, but follow a similar structure (Figure 5C). The predicted constant curvature values $\Phi_{s}$ and *k*_s_ are evaluated against the target values $\Phi_{t}$ and *k*_t_. For *k*, the predicted and target values are directly compared. For Φ, instead of a direct comparison, the relative distance between $\Phi_{s}$ and $\Phi_{t}$ is computed and evaluated against a predefined threshold. If the target value is larger than the sensor value, then the individual actuator is pressurized; conversely, if the target value is smaller than the sensor value, then the actuator pressure is decreased. The pressure step size is defined by a tip distance dependent function, which increases the step magnitude when the sensed robot position is far from the target and progressively decreases as the robot approaches the desired location. The loop pauses when the sensor value matches its respective target value, and the control process concludes once both $\Phi_{s}$ and *k*_s_ values reach their corresponding targets. This straightforward control methodology successfully moved our robot to reach three target locations in space, all spanning different $\Phi_{t}$ and *k*_*t*_ values (Figure 5A). The robot's path to reach the desired locations is shown by the respective true *x*, *y*, and *z* tip positions, accompanied by the sensor's predicted tip values calculated using Equation (3). The average tip position error between the predicted and true values for all three tests is 0.58 mm. The relative pressure changes of the three actuators are also shown in Figure 5A, confirming that the control method was able to select and adjust the pressure of the two dominant actuators out of the total three, based on the target values, to reach the desired point in the robot workspace. To emphasize the precision of the shape‐sensing system, the pressure steps were deliberately kept small, allowing for precise actuation and demonstrating the sensor's ability to guide the robot to target locations with high positional accuracy.

### 2.6 Multiple Point Following and External Force Validation

**FIGURE 6:**
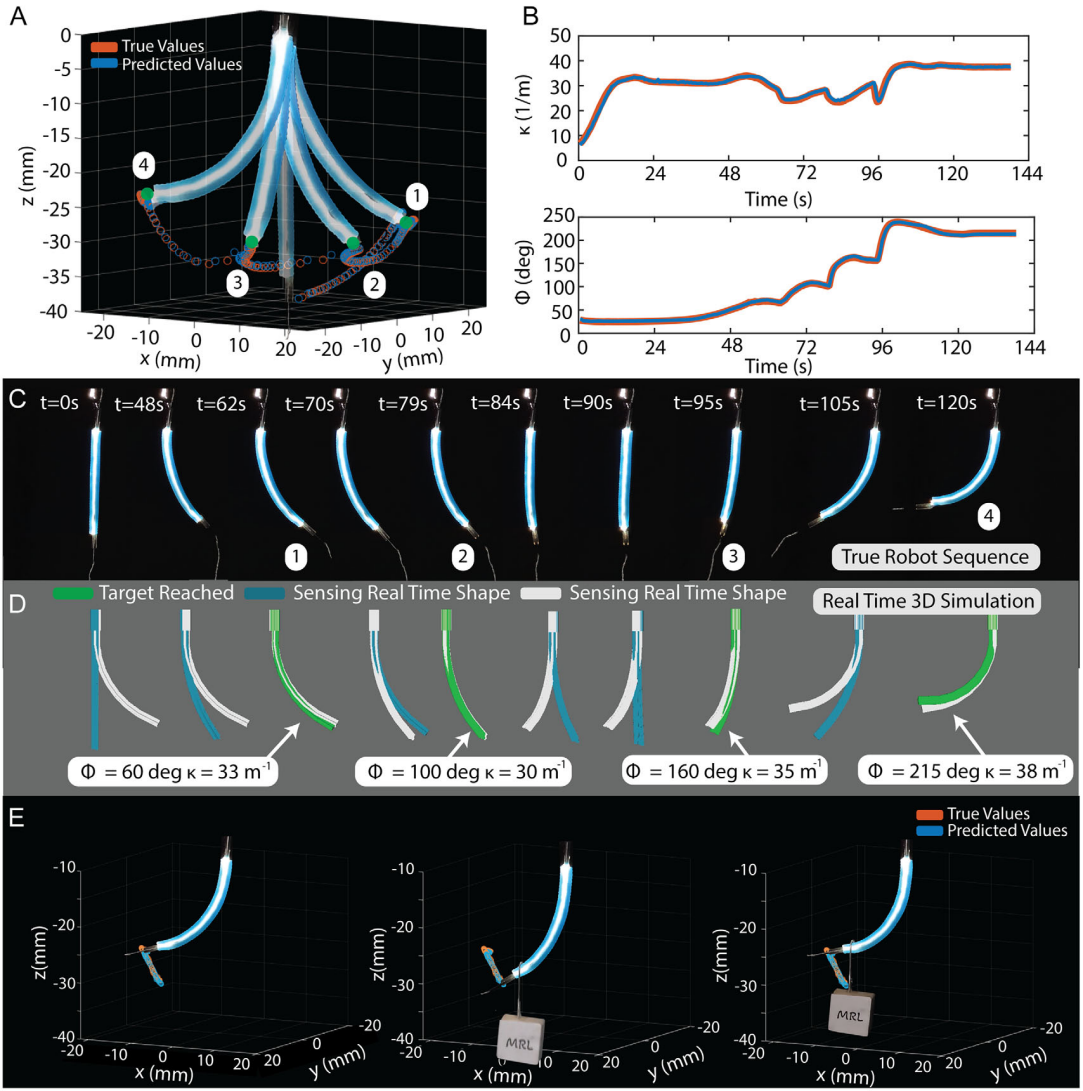
Path following and external force testing. (A) The resulting 3D plot for the true and predicted tip position during the path following test with the robot superimposed at each target location is shown. (B) The plots for the true and predicted Φ and *k* during the navigation to the desired points are plotted with respect to time. (C) The snapshots of the real robot moving through space in a sequence are depicted. (D) Real‐time 3D‐rendered simulation of LUMOS's response. The blue robot represents the sensor's real‐time predicted shape, while the white robot indicates the target configuration. The blue rendering transitions to green upon successful alignment with the target shape. (E) Plot showing the external force test with the true and predicted shape‐sensing tip location plotted in 3D with the robot superimposed. Three steps are depicted: when the robot reaches its initial target location (Left), when the external weight is applied (Middle), and when it returns to its original position (Right).

Further extending the demonstrated capabilities of LUMOS in our soft robot, we demonstrate additional functionalities utilizing the closed‐loop control methodology. As an example of a more realistic utilization of a soft continuum robot, we have the robot move to four target positions in succession to create a path across a wide range of $\Phi_{t}$ and *k*_t_ values (Figure 6A). The robot successfully reached all four points in 3D space, each marked with a green dot labeled 1 through 4, switching between different dominant actuators to accomplish this. The true and predicted tip values were recorded and plotted (Figure 6A), showing the accuracy of the shape of the robot during this navigation, having an average tip error of 0.64 mm for the entirety of the experimental procedure. Additionally, the plots showing the real and predicted Φ and *k* values confirm the target values were reached along the path (Figure 6B), going from a Φ value of 60° to 215° and spanning curvatures from 33 to 38 m^−1^ having an average error between the true and predicted values of 1.3° for Φ and 0.4 m^−1^ for *k*. Snapshots of the physical device during the navigation test are presented at the target point time steps (Figure 6C). The time between reaching the first target location and the last was 54 s, taking an average of 19.3 s between points after the first target was reached. A majority of the total time for the entire test was taken to initially pressurize the actuators to initiate the movement of the robot from its base straight configuration. This is accompanied by the real‐time 3D‐rendered simulation of the device developed in Python (Panda 3D) (Figure 6D). The real‐time simulation view shows the current predicted output of the robot in blue and the target robot's position in white, which changes to green when the robot reaches its target location. This enables direct visualization of the robot's sensor output and task execution without the need for external visual feedback. This test was conducted for the four points in 3D space and also for ten points, as shown in Supporting Movie S5.

Many robotic applications require the ability to maintain a specific position in space, even when subjected to external loads that could potentially come from grasping an object or interacting with a moving environment, ensuring stability and precision in various operational conditions. In Figure 6E, we show the robot and soft sensor's capability to hold its position in 3D space and do so while compensating for an external load on the robot's tip. The robot was first actuated, using the closed‐loop control methodology, to reach a specific point in the workspace and hold its position (Figure 6E). Proceeding this, an external weight made of laser‐cut wood weighing 0.5 g, which is 1.3 times heavier than the robot's soft distal tip weighing 0.38 g, was placed on the end of the robot using tweezers. The resulting shape‐sensing response of the robot updates in real‐time (Figure 6E Middle), and in response, the two dominant pneumatic actuators are increased in pressure to actuate the robot to return to the original target location (Figure 6E Right). The accuracy between the predicted and true tip position from the moment the weight was placed onto the robot tip is 1.06 mm, showing the ability to handle external loads both in sensing and control, and return to its original position. This demonstration is accompanied by a real‐time 3D‐rendered simulation and is seen in Supporting Movie S6.

To further characterize the robustness of how external weight affects LUMOS's response, a systematic loading test was performed. Specifically, the robot was incrementally loaded at its tip with weights up to 10.46 g (27.53 × the robot's weight), with the resulting analysis shown in Supporting Figure S4. As illustrated in Supporting Figure S4B, the corresponding plot reports the tip error for each specific weight tested. The prediction error increased with moderate loads (peaking at 4.76 × robot weight) but decreased at higher loads, where the robot was pulled into a straighter configuration. Remarkably, the sensor remained functional and accurate up to the maximum applied load, highlighting the robustness of the sensing approach.

### 2.7 Autonomous In Vitro Navigation

**FIGURE 7:**
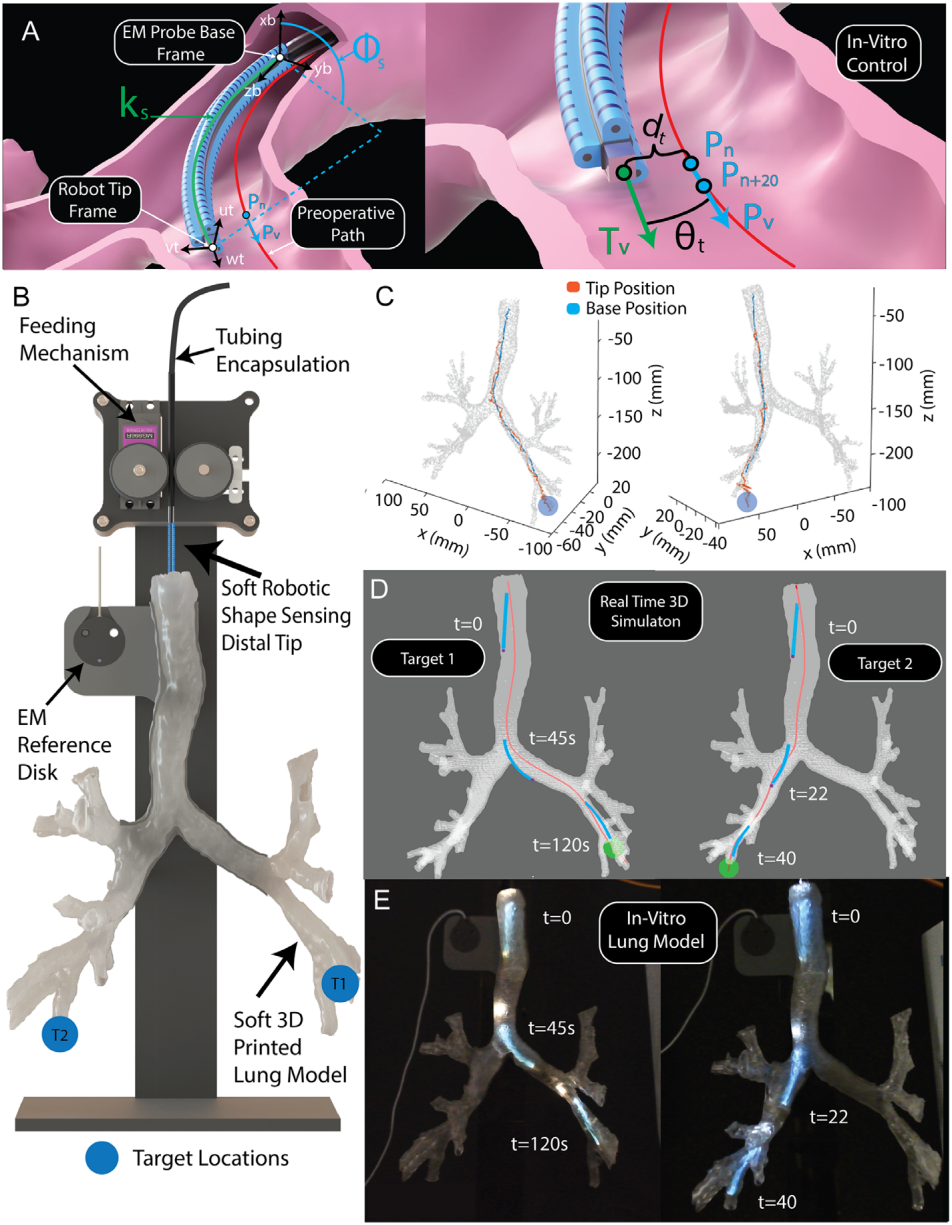
Autonomous in vitro control. (A) The control diagram for the autonomous navigation within the in vitro environment. Real‐time shape predictions ($\Phi_{s}$, *k*_s_) from LUMOS are compared to target values ($\Phi_{t}$, *k*_t_) derived from the closest point on the preoperative path *P*_*n*_. The two dominant actuators are adjusted until the tip to path distance (*d*_t_) and tip to path angle (*θ*_t_) fall within predefined thresholds, at which point insertion is performed. (B) The in vitro experimental setup including the soft 3D‐printed lung model, the EM reference disc for robot localization within the lung, the feeding mechanism, the two predefined target locations on the left and right sides of the lung environment, and the insertion point for the soft robotic platform. (C) The autonomous navigation shows the robot base position and predicted tip position for navigation of reaching target one into the lower right lung periphery and target two into the lower left lung periphery. (D) The real‐time 3D simulated view of the robot at three distinct time steps during the navigation, all superimposed on the same plot. The blue 3D model displays the robot's real‐time shape at these time steps, where the green sphere at the end of the lung indicates that the robot has navigated to the target location. (E) Three snapshots from recorded experimental videos show the physical robot navigating through the in vitro lung environment, all superimposed within the same image to illustrate its progression toward the target locations.

To illustrate the potential impacts of LUMOS, we have extended the demonstrated closed‐loop control and omnidirectional bending functionalities to a more realistic, clinically relevant environment (Figure 7A). To this end, we created a true‐to‐size, soft 3D‐printed lung phantom by segmenting patient computed tomography (CT) scan data in 3D Slicer to generate a digital airway model, which was subsequently fabricated using a flexible resin on a high‐resolution 3D printer (Formlabs Form 4) that replicates the bronchial tree anatomy (Figure 7B). The full design and experimental setup are described in detail in the Materials and Methods (In Vitro Testing Setup and Preoperative Planning). We picked the bronchial tree for navigation due to its complex environment to navigate, involving sharp bends and inner wall diameters that decrease in size as one traverses further into the lung environment [55, 56, 57–58]. We demonstrated autonomous navigation with our platform to two desired target locations in the lung phantom, one on each side of the periphery of the lung (Figure 7B). A control method that leverages the real‐time shape‐sensing output and omnidirectional bending capabilities was developed to enable the robot to follow preplanned preoperative paths. The base position of the robot ***P***_b_ is defined by the EM probe at the soft actuated distal tips base (Figure 7A). The real‐time shape of the robot ($\Phi_{s}$, *k*_s_) is predicted using the proposed trained neural networks mentioned in Section (Shape‐Sensing Characterization and Validation) and the robot tip position ***P***_t_ is calculated using Equation (3). A K‐D tree algorithm is used to calculate the closest point on the preoperative path to the tip position of the robot, ***P***_*n*_. A relationship between the robot's base position ***P***_b_ and the closest point on the path ***P***_*n*_ is established, and a target $\Phi_{t}$ and *k*_t_ is calculated using Equation (1) and Equation (2). The closed‐loop control logic is then employed to actuate the robot to reach these specific target constant curvature arc values by adjusting the pressure of the two dominant actuators. To monitor the relationship between the predicted shape of the robot in relation to the preoperative path, we have defined two variables. The first is ***d***_*t*_, which is the distance between the robot tip position ***P***_*t*_ and the closest point on the path ***P***_*n*_, and the second variable is *θ*_*t*_, which is the angle between the robot's normal tip vector ${\vec{T}}_{v}$ and path vector ${\vec{P}}_{v}$ (Figure 7B Right). The vector ${\vec{P}}_{v}$ is defined as a vector between ***P***_*n*_ and ***P***_*n* + 20_, which is twenty points further down the preoperative path. During navigation, the robot decides to conduct insertion based on the distance ***d***_t_ and the angle *θ*_t_. The tolerance distance ***t***_d_ and angle ***t***_θ_ are predefined and set to 5 mm and 28°, respectively. If the shape of the robot predicts values that are within these tolerances, then closed‐loop control is paused, and the robot is inserted using the feeding mechanism at the top of the in vitro setup, the design shown in Figure S3. If these tolerances are not met, the closed‐loop control logic will keep pressurizing and depressurizing the two dominant actuators until it has done so. The key system‐level performance parameters, including closed‐loop control speed, the sampling rate of LUMOS, the EM probe tracking rate, and the update rates of the pump command and neural network, are summarized in Supporting Table S4.

Utilizing this control method, we autonomously reach the left and right side targets within the lungs’ bronchial tree (Figure 7C–E). During these tests, our proposed continuum robot and embedded LUMOS were able to smoothly navigate through the path while making the required bends and alignments to move down the correct bronchial tree branches. The robot base position and estimated tip positions during both navigations are plotted in Figure 7C, showing the capability of the shape‐sensing response to stay within the bronchial tree environment and have sufficient accuracy to guide the robot down the desired preoperative paths even when experiencing external forces when interacting with the phantom lungs luminal walls. During navigation, the robot's real‐time shape‐sensing response is used to render its predicted shape and position in 3D within a simulated lung environment. This visualization, shown in Figure 7D, enables intuitive monitoring of the robot's movement through the anatomy. This capability is particularly valuable in a surgical setting, where precise shape visualization provides surgeons with essential visual feedback to understand the real‐time state of the robot. Figure 7E depicts snapshots of the experimental setup and the robot shown through the clear soft phantom lung at the same time stamps as shown in the simulation. The total time to reach the bottom‐right target location (Target 1) is 120 s, as it requires deploying larger curvatures, resulting in longer navigation. In contrast, reaching Target 2 takes 40 s. While the shown snapshots illustrate the overall shape prediction during navigation, Supporting Movie S7 more effectively highlights the sensor's real‐time shape‐sensing response and performance as the robot navigates to two target locations within the lung. This real‐time demonstration also emphasizes the importance of the sensor's miniaturization; by reducing its footprint, the overall robot size is minimized, enabling successful navigation through tight, hard‐to‐reach environments.

## 3 Conclusion

In this study, we present LUMOS, a 1.25 mm soft optical sensor capable of full three‐dimensional shape reconstruction. This is achieved through a color‐shifting response captured from a single transmitted light signal. LUMOS incorporates a thin film color‐tuning technique within its central soft optical core, enabling a resulting curvature and direction‐dependent RGB response while maintaining a minimal overall footprint. A flexible mirror is leveraged at the distal tip of the device to reflect light transmitted through a thin outer waveguide back through the color‐tuned core, where the signal is subsequently captured. This mirror allows both signal delivery and retrieval to occur entirely at LUMOS's base, eliminating the need for looped optical paths or distal electrical components, thereby enhancing the sensor's scalability and miniaturization. As a result, the design simplifies integration into soft robots due to the reduced spatial constraints and single signal monitoring, paving the way to seamless implementation across diverse soft robotic platforms.

To assess the ability of the color‐shifting response to accurately predict a robot's shape, LUMOS was embedded into a 3.2 mm diameter soft robotic platform. The fully soft continuum robot, equipped with three pneumatic actuators that enable omnidirectional bending, was actuated throughout its 3D workspace to collect RGB data corresponding to a wide range of shape configurations. This dataset was then used to train feed‐forward neural networks capable of predicting the robot's real‐time shape, achieving an average tip position accuracy of 0.47 mm across its entire workspace.

In an unconstrained environment, the robot successfully performed shape‐guided closed‐loop target point navigation by actively varying actuator pressures in response to real‐time 3D shape‐sensing feedback from LUMOS. Leveraging this control, the robot successfully followed multiple points on a predefined 3D path, exemplifying a more realistic application of the closed‐loop architecture. To assess LUMOS's ability to compensate for external force disruptions, a load compensation test was conducted by applying an external load equivalent to 1.3 times the robot's own mass to its tip. The real‐time shape feedback allowed the system to detect the disruption to the shape and actively compensate to return to the original target location. Finally, paired with an external feeding mechanism, the robot autonomously navigated a soft, anatomically realistic in vitro lung phantom, using shape‐sensing feedback in real‐time to follow a preoperative path within the complex airway geometry. This environment poses significant challenges due to its branching structure, external contact loads, sharp bends, and progressively narrowing inner diameters, demonstrating LUMOS's ability to support shape‐guided control in constrained and complex environments. If advanced toward clinical translation, additional factors would need to be considered, including the ability of the control system to account for breathing‐induced lung motion, divergence between preoperative computed tomography data and the live patient environment, and the irregular, nonconstant curvatures present when interacting with compliant tissue. A potential future clinical direction would be to couple LUMOS shape‐sensing with real‐time visual feedback from a miniature camera to create a more robust and reliable navigation system.

In conclusion, this work presents a compact, fully soft, and highly information‐dense sensing approach for real‐time shape reconstruction in soft robotic systems. The thin film color‐tuning method and thin film mirror enable curvature and direction‐dependent signal encoding within a single soft optical waveguide, thereby avoiding the need for embedded electronics or complex routing, which addresses key challenges in size, integration, and manufacturing complexity for the utilization of soft optical WGs in soft robots. Beyond the demonstrations in this work, our proposed sensing approach provides a flexible foundation for expanding shape‐sensing resolution and functionality. In future work, the thin film color‐tuning method could be adapted through spatially varied or layered film configurations, enabling more complex shape detection and localized or directionally sensitive responses with minimal increase in sensor size. In addition, the use of pigments responsive to nonvisible wavelengths of light, such as ultraviolet or near‐infrared, could expand the tuning space and unlock more complex spectral encoding, further enriching the information available from a single embedded sensor.

## 4 Materials and Methods

### 4.1 Soft Thin Input WG Manufacturing

**FIGURE 8:**
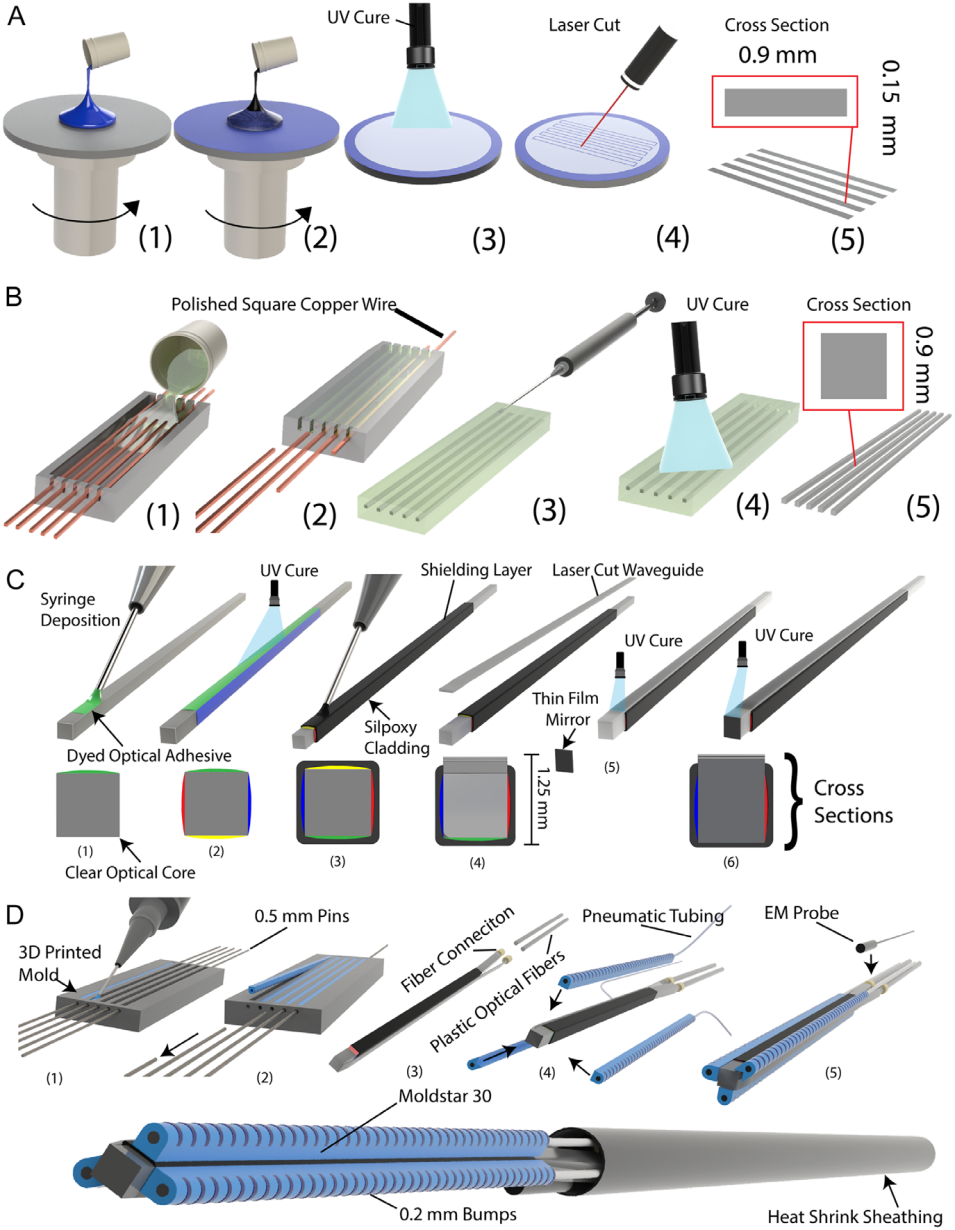
Overview of the manufacturing steps for LUMOS and integration into the soft robot. (A) Manufacturing process for the thin input WGs including the spin coating of both the silicone and optical adhesive in steps (1–2), the UV curing and laser cutting of the thin adhesive sheet in steps (3–4) and finally the end result and cross‐section of the thin outer WG (5). (B) Manufacturing process for the clear shape‐sensing core including the molding process using square copper wire in (1–2) the optical adhesive injection and UV curing in (3–4), and the final cross‐section result shown in (5). (C) The manufacturing process of LUMOS, highlighting the thin film deposition of the pigmented NOA 65 (1–2), the deposition of the cladding material (3), the connection and bonding of the thin outer WG (4–5), and the attachment of the thin film mirror at the sensor's tip (6). (D) Manufacturing process for the robot assembly of embedding LUMOS, showing the molding method and pin removal for the soft actuators (1–2), the fiber connection to the base of LUMOS (3), the actuator connection, EM probe placement, and heat‐shrink sheathing encapsulating all tubing and fiber connections (4–5).

The manufacturing steps for LUMOS and its integration into the soft robot are shown in Figure 8. The first step is making the thin outer WG (Figure 8A). First, MoldStar 30 is spin‐coated on a circular sheet of acrylic and cured in an oven at 70°C (Figure 8A(1)). After this, uncured NOA 65 (25 g) is spin‐coated at 300 rpm for 30 s on top of this silicone (Figure 8A(2)). During spinning, the resulting sheet of NOA 65 is cured using a UV light (Uvitron SunSpot 2), leaving a large, thin sheet of cured NOA 65 on the surface of the silicone (Figure 8A(3)). A CO_2_ Laser (VLS6.75 Universal Laser System) is then used to cut out (0.9 by 0.15 mm) into NOA 65 strips, allowing us to make 40 strips from one NOA 65 sheet (Figure 8A(4,5)). These strips are then removed from the acrylic sheet and washed in a bath of isopropyl alcohol to remove any residue from laser cutting.

### 4.2 Soft Optical Core Manufacturing

In order to make the clear square soft optical core, we first polish 0.9 × 0.9 mm copper wire to a mirror finish. These wires are then placed into a 3D‐printed mold shown in Figure 8B. Ecoflex 30 is then poured into the 3D‐printed mold and cured in an oven at 70°C (Figure 8B(1)). The copper wire is then pulled out of the mold, leaving a smooth square cavity in its place (Figure 8B(2)). Clear NOA 65 is injected into these cavities using a syringe and cured using UV light (Figure 8B(3,4)). Finally, the clear cores with a cross‐section of 0.9 mm are demolded from the Ecoflex 30 shell to be used in color tuning (Figure 8B(5)).

### 4.3 Colored Thin Film Manufacturing, and Sensor Assembly

NOA 65 (2 ml) is mixed with the dyes in C2 concentrations (Supporting Table S1). The amount of dye used was as follows: red (DecorRom Concentrated UV Resin Colorant Rose, 0.048 g), blue (EPODEX Transparent Blue Drop‐in Dye, 0.052 g), green (DecorRom Concentrated UV Resin Colorant Olive, 0.200 g), and yellow (DecorRom Concentrated UV Resin Colorant Amber, 0.060 g). Then the mixture is deposited by using a syringe and a 25 gauge needle, putting one color on each side of the soft optical core at the distal 38 mm and cured using a UV light (Figure 8C(1,2)).

For the application of the cladding, we mix Sil‐Poxy and black pigment dye (Silc Pig) with 50% of solvent by mass (DOWSIL OS‐2 Silicone Solvent) to lower the viscosity of the Sil‐Poxy, using a Thinky ARE‐310 Mixer. This mixture is then placed into a syringe, deposited on all sides of the optical core (Figure 8C(3)), and cured at 70°C. To attach the thin outer WG, Sil‐Poxy is deposited onto the surface of one of the sides of the black cladding, adding 0.07 mm thickness to the outer dimension. Then, the thin WG is placed onto this adhesive and pressed against the WG's surface, ensuring that no adhesive is applied to the exposed WG core section (Figure 8C(4)). A drop of NOA 65 is placed on the color‐tuned core's exposed surface, and the thin outer WG is laid on top of this drop. The NOA 65 disperses between the core and WG to cover the entire exposed area and is then cured using UV Light (Figure 8C(5)).

Finally, the laser‐cut mirror is attached to the color‐tuned cores’ end face. A drop of NOA 65 is placed on the face and the mirror is then placed on top of this adhesive, resulting in the mirror lying flush to the surface, and UV‐cured, fully bonding it to the WG tip (Figure 8C(6)). The full Manufacturing and assembly of LUMOS is shown in Supporting Movie S2.

### 4.4 Actuator Manufacturing and Robot Assembly

To create the soft, miniaturized actuators, a 3D‐printed mold (Formlabs Form 4) containing the actuator cavities was fabricated, utilizing 0.5 mm pins to form the actuation channel (Figure 8D(1)). MoldStar 30 is poured into each mold and then cured at 70°C. The pins are then demolded, and the actuators are pulled from the 3D‐printed mold (Figure 8D(2)). Before connecting the actuators to the soft color‐tuned sensor, the sensor is connected to two plastic optical fibers using tubing that has an inner diameter of 0.78 mm and an outer diameter of 1.5 mm, and the fibers are pushed into that tubing and UV cured to the input and output of the soft optical waveguide (Figure 8D(3)). The actuators are then attached to the shape‐sensing core using Sil‐Poxy; two are attached to the flat sides of the square core, and the last is attached to the edge of one of the resulting exposed sides (Figure 8D(4)). The EM probe is attached to one side of the base of the shape‐sensing core using Sil‐Poxy (Figure 8D(5)). Finally, the tubing used for actuation is attached to the input side of the actuators, and all the tubing, fibers, and EM probe wiring are fed through the soft encasement tubing (Figure 8D bottom). The soft encasement tubing (Sumitomo B2 black spl heatshrink) was stretched until the desired 3.2 mm diameter was reached and fed around the robot up to the base of the soft tip.

### 4.5 Sensor Calibration and Closed‐Loop Control Testing Setup

The testing setup for the closed‐loop control and sensor calibration is shown in Figure S3A. A laser‐cut acrylic stand and a 3D‐printed robot mount were used for these tests. The robot mount was designed to only grip the soft encasement tubing around the device and have no contact with the robot's distal tip. Furthermore, the acrylic stand was made tall enough for the EM probe wire to hang freely and not affect the calibration and validation results during testing. To attach the EM probe at the tip of the device to monitor the tip location, a small tube was connected to the robot's tip, and the EM probe was pushed with a friction fit. A custom sensor box was designed and implemented to both send and receive the color light signals for this calibration testing setup. Specifically, an LED (XLamp CXA2520 White) was used for the input, and the RGB color sensor (Adafruit TCS34725) was used for the color output retrieval (Figure S3B). Custom 3D‐printed connectors were made and slotted around the input and output optical fibers to ensure consistent and repeatable connections when moving the robot or unplugging it for transportation. Additionally, a fiber mount system for the input color monitoring side was made to locate the fiber in the center of the color sensor to ensure a consistent, strong signal response.

### 4.6 In Vitro Testing Setup and Preoperative Planning

For the in vitro testing setup, a virtual lung environment is obtained through initial CT imaging of a patient's lungs (anonymous CT scans were provided by a collaborating clinician). The CT images are segmented using 3D Slicer to obtain a virtual 3D model of the patient's airways. The lung model was then 3D‐printed using a soft elastic resin (Elastic 50A Resin V2 Form 4) in three separate sections and connected using Sil‐Poxy. In addition, a 3D‐printed mount was made that holds the lung in place using Sil‐Poxy to make sure it does not shift or move during testing. On the mounting system, a cavity is made to place a reference EM disk to locate the robot within the 3D‐printed lung model environment, which can be seen in Figure 7B. Additionally, a custom feeding mechanism for our device was manufactured that uses a single servo and two friction wheels to be able to feed our robot into the lungs (Figure S2).

The centerlines of preoperative paths within the lung model are found using the Vascular Modeling Toolkit module within 3D Slicer. The resulting 3D lung model and centerlines are exported into MATLAB and converted into point clouds. The centerline point cloud is then used as a graph through which the robot can navigate. The $A^{*}$ algorithm is then used to plan the path from the beginning of the trachea into each target. Finally, the planned path is smoothed using the “rloess” method in the MATLAB Smooth function and assigns a lower weight of 0.2 to outliers in the regression to remove any harsh, jagged edges that could potentially affect the autonomous control.

### 4.7 Neural Network Implementation

To decipher the signals used in neural network training, the RGB values are obtained from the color sensor and normalized by dividing them by 255 to ensure they fall between 0 and 1. In addition, the Φ values are encoded using sin (Φ) and cos (Φ), which removes a discrepancy at the wraparound point between 0° and 360°. This discrepancy can cause errors when training our networks; thus, it was mitigated. The collected *k* values are unchanged when used in the model. The first network takes normalized RGB signals as input and predicts the bending orientation Φ. The second network takes the same RGB inputs and predicts curvature *k*. We trained two separate networks to independently optimize performance on each prediction task. The dataset was augmented by generating noisy variants to simulate measurement variability and enhance the robustness of the networks, thereby allowing them to generalize more effectively to experimental data. Both networks are feed‐forward neural networks that use Bayesian regularization as the training algorithm, with the mean squared error (MSE) as the loss function. The dataset was randomly shuffled and split into independent training (90%) and unseen validation data (10%) sets to provide an unbiased evaluation of network performance. Each of the two trained networks is composed of three hidden layers, comprising 30 neurons in the first layer, 20 in the second, and 10 in the third. The number of epochs for the first network is 200, while the second network has 300 epochs. After training, an encoded predicted Φ value is decoded using arctan 2(sin(Φ),cos(Φ)). For the second neural network, we use just the output curvature for our prediction. For both networks, a tight convergence criterion was used; this was feasible due to the exceptionally clean and consistent nature of the dataset, which allowed the networks to map the data without overfitting.

## Supporting Information

Additional supporting information can be found online in the Supporting Information section. **Supporting Fig. S1:** Experimental setup for evaluating the optical sensor response during bending. **Supporting Fig. S2:** Feeding mechanism design. **Supporting Fig. S3:** Calibration Testing Setup and Control Box. **Supporting Fig. S4:** Incremental Load Testing of the Robot and resulting LUMOS Response. **Supporting Table S1:** Percentage concentration of dye in NOA 65 optical adhesive thin films for each specific dye color. **Supporting Table S2:** The resolution of each thin film dye with regard to the dB loss over curvature. **Supporting Table S3:** The Signal to Noise Ratio across colored dyes and concentrations C1–C3. **Supporting Table S4:** System‐level performance characteristics. **Supporting Table S5:** Comparison of shapesensing approaches. **Supporting Movie S1:** Color Shifting Thin Film Waveguide Core Response. **Supporting Movie S2:** Manufacturing Overview of LUMOS. **Supporting Movie S3:** Optical Color Signal Response as a Result of Bending the Omnidirectional Robot. **Supporting Movie 4:** Real‐Time Shape‐Sensing Response of the Robot as it Moves in 3D Space and with external disturbances. **Supporting Movie S5:** Closed‐Loop Control to Multiple Points in Space. **Supporting Movie S6:** External Force Control Test. **Supporting Movie S7:** In Vitro Autonomous Navigation.

## Conflicts of Interest

The authors declare no conflicts of interest.

## Supporting information

Supplementary Material

## Data Availability

The data that support the findings of this study are available from the corresponding author upon reasonable request.
