# Supplementary material for: Enabling Real‐Time Shape‐Sensing in Soft Robots via a Miniaturized, Single‐Signal, Color‐Tuned Soft Optical Sensor
Source: Adv Robot Res. 2025 Nov 8;2(1):e202500124. doi: 10.1002/adrr.202500124 (PMC13238374; doi:10.1002/adrr.202500124)
Supplement: Supplementary file 1 — Supplementary Material [file ADRR-2-e202500124-s001.zip › Supporting_Information_ARR.pdf]

Supporting Information

Enabling Real-Time Shape Sensing in Soft Robots via a Miniaturized, Single-Signal, Color-Tuned Soft Optical Sensor

Frank Juliá Wise[1*] *Yitong Lu*[1] *Daniel Van Lewen*[1] *Riley Applebaum*[1] *Bianca Andrada*[1] *Johnathan Reamer*[1] *Sheila Russo*[1*]

Table of Contents

- Tables S1 to S5 .... Page 2
- Figures S1 to S4 .... Page 3
- Captions for Movies S1 to S7 .... Page 7

Tables:

Table S1: Percentage concentration of dye in NOA 65 optical adhesive thin films for each specific dye color.
Table S2: The resolution of each thin film dye with regard to the dB loss over curvature.
Table S3: The Signal to Noise Ratio across colored dyes and concentrations C1–C3.
Table S4: System-level performance characteristics.
Table S5: Comparison of shape sensing approaches.

Figures:

Figure S1: Experimental setup for evaluating the optical sensor response during bending.
Figure S2: Feeding mechanism design.
Figure S3: Calibration Testing Setup and Control Box.
Figure S4: Incremental Load Testing of the Robot and resulting LUMOS Response.

Movie Captions:

Movie S1 Caption: Color Shifting Thin Film Waveguide Core Response
Movie S2 Caption: Manufacturing Overview of LUMOS
Movie S3 Caption: Optical Color Signal Response as a Result of Bending the Omnidirectional Robot
Movie S4 Caption: Real-Time Shape-Sensing Response of the Robot as it Moves in 3D Space and with external disturbances
Movie S5 Caption: Closed-Loop Control to Multiple Points in Space
Movie S6 Caption: External Force Control Test
Movie S7 Caption: In-Vitro Autonomous Navigation

Table S1: Dye Concentration. Percentage concentration of dye in NOA 65 optical adhesive thin films for each specific dye color.

Dye Color	C1 (%)	C2 (%)	C3 (%)
Red	0.05	2.40	4.50
Blue	0.05	2.60	4.00
Yellow	0.10	10.00	20.00
Green	0.10	3.00	11.50

Table S2: Dye Curvature resolution. The resolution of each thin film dye with regard to the dB loss over curvature.

Dye Color	C1 (dB / m^{-1})	C2 (dB / m^{-1})	C3 (dB / m^{-1})
Red	0.0118	0.0485	0.0688
Blue	0.0449	0.0877	0.0973
Yellow	0.0187	0.0249	0.0379
Green	0.0133	0.0462	0.0743

Table S3: Signal-to-Noise Ratio (SNR) across dyes and concentrations C1–C3.

Dye Color	C1 (SNR)	C2 (SNR)	C3 (SNR)
Red	417.55	78.94	14.30
Blue	94.35	130.08	30.15
Yellow	140.17	47.30	11.23
Green	198.80	130.88	3.90

Table S4: System-level performance characteristics.

**Closed-loop Speed (ms)**	234.55
**LUMOS Sampling Rate (ms)**	50.21
**Aurora Sampling Rate (ms)**	24.93
**Pump Command Update Rate (ms)**	30.56
**Neural Network Update Rate (ms)**	24.23

Table S5: Comparison of shape sensing approaches.

**Shape-Sensing Method**	**Footprint**	**Compliance**	**Readout Complexity**	**Notes / Limitations**
FBG (multi-core)	submillimeter scale	Rigid (silica glass)	High	High accuracy but fragile; limited bending radius; costly equipment required.
Multi-sensor soft systems	Multiple millimeter scale sensors	Semi-soft (depending on integrated sensors)	Medium-High	Enables curvature sensing but bulky; difficult to miniaturize; reduced compliance due to multiple sensor designs.
**LUMOS**	**1.25 mm cross section**	**Fully soft**	**Low**	**Fully compliant and soft; tolerant to large curvatures; single optical channel; miniaturizable and cost effective; easily embedded into soft robotic platforms.**

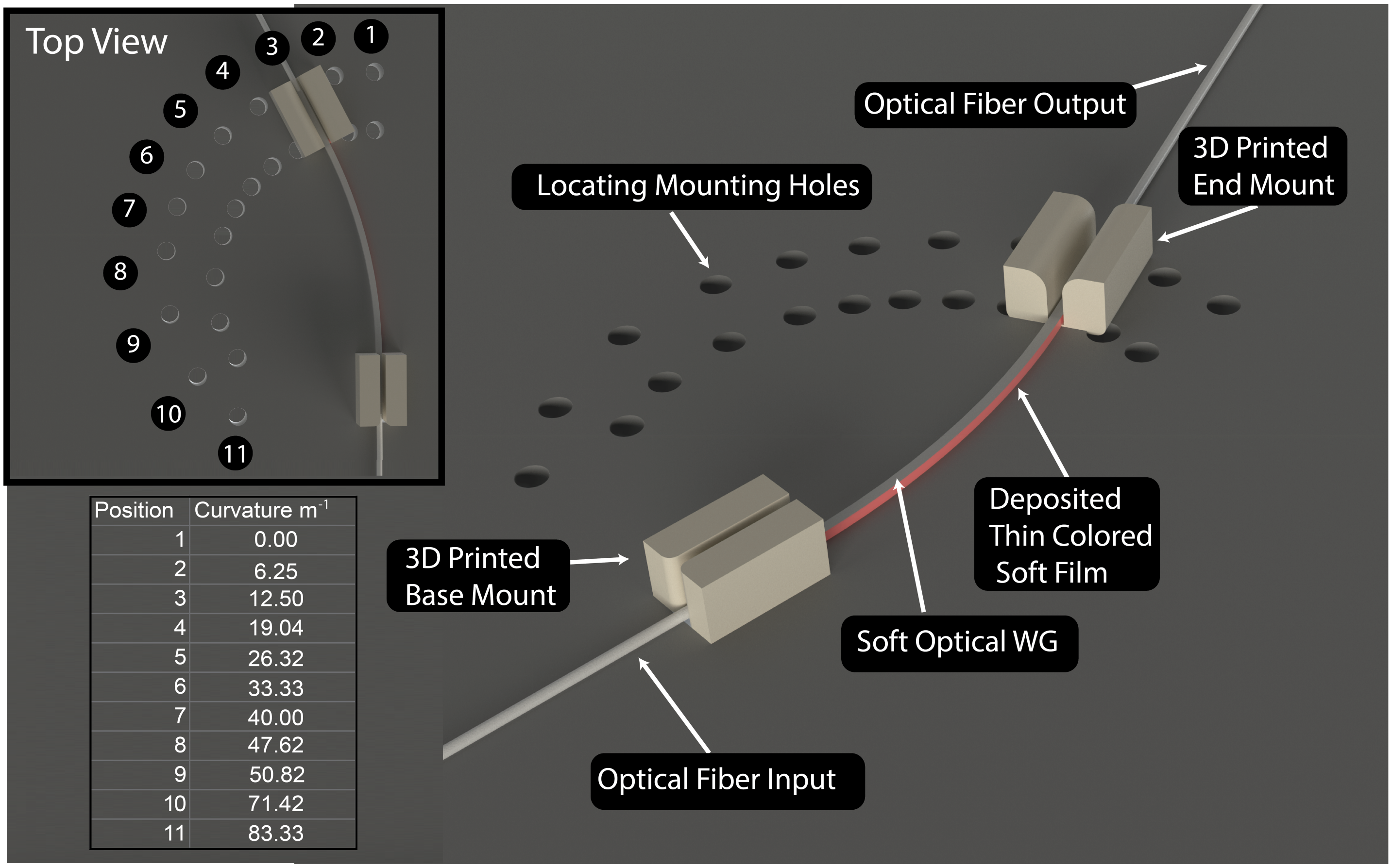

Position	Curvature m^{-1}
1	0.00
2	6.25
3	12.50
4	19.04
5	26.32
6	33.33
7	40.00
8	47.62
9	50.82
10	71.42
11	83.33

Figure S1: Experimental setup for evaluating the optical sensor response during bending. The sensor is mounted between two 3D printed mounts holding the sensor in a constant curvature manner in space. The mounting holes in the testing setup locate the 3D printed end mount to different curvature values, labeled 1 through 11, corresponding to curvature values listed in the bottom-left table. The outer thin film layer is also visible on the opposite side of the bend, shown in red. Fibers are attached to the base and end of the sensor core to send and receive light signals during testing.

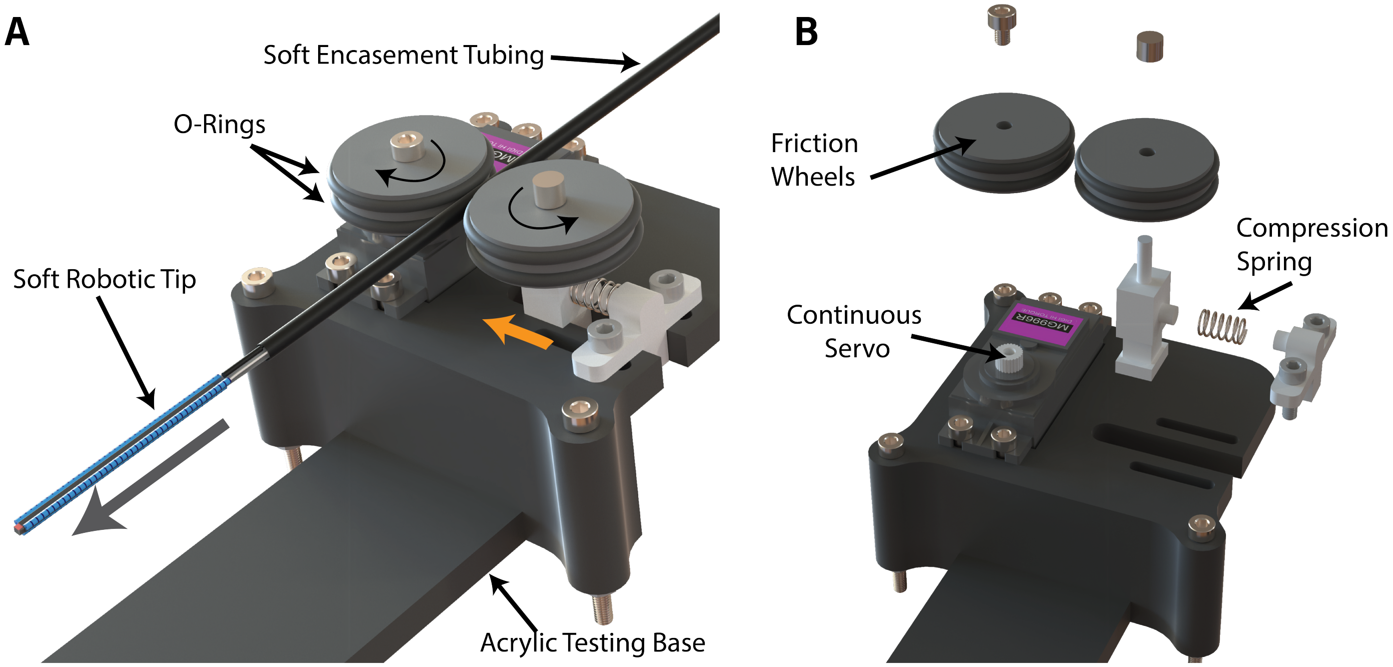

Figure S2: Feeding mechanism design. **(A)** Fully assembled device in operation. The two friction wheels are pressed against the soft encasement tubing, with consistent contact maintained by a compression spring that provides the necessary force for engagement. The continuous servo that is attached to the friction wheels spins the wheel, thus feeding the robot forward, further down the lung environment. **(B)** The exploded view of the components of this assembly.

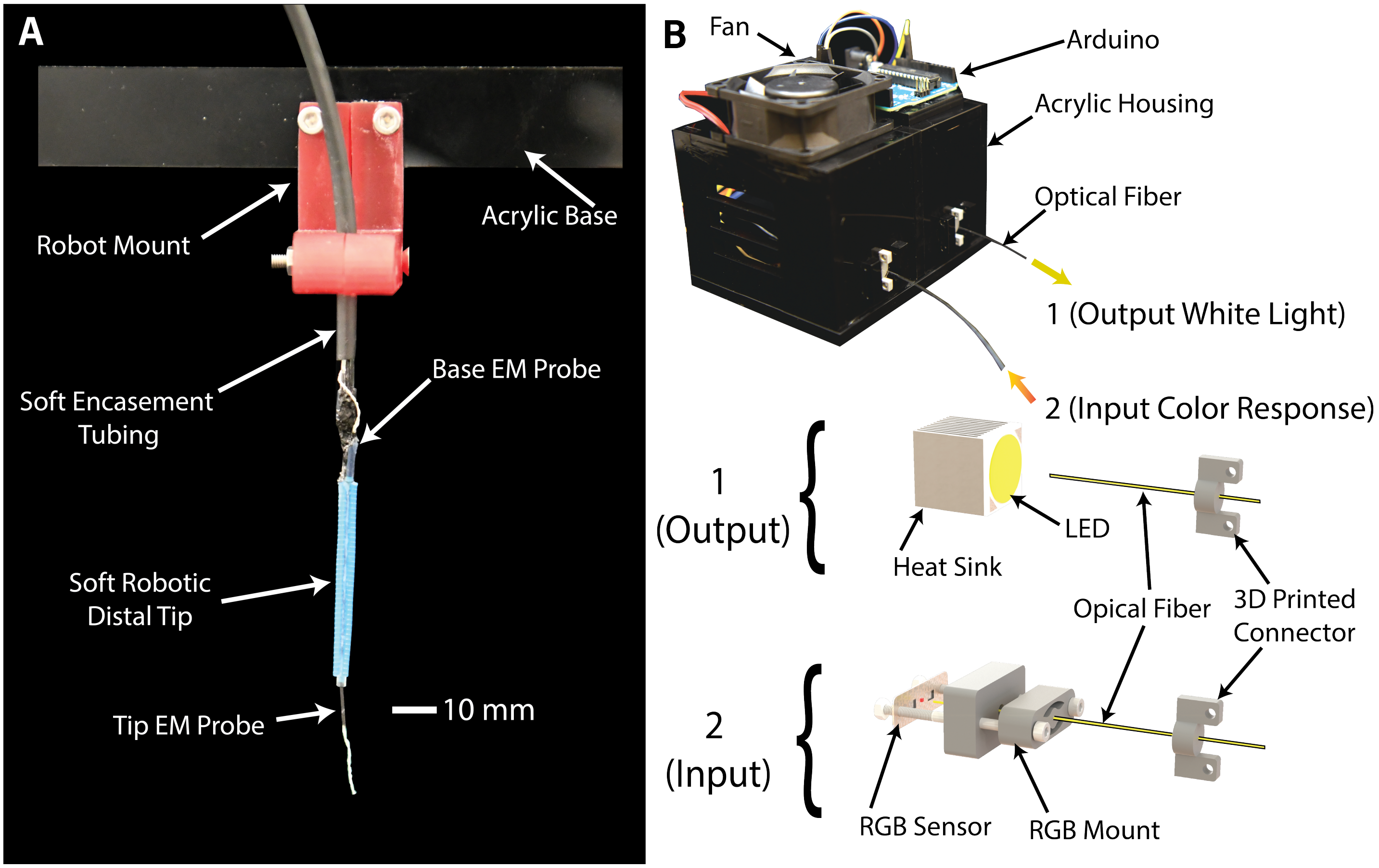

Figure S3: Calibration Testing Setup and Control Box. **(A)** The free space testing setup, including the Acrylic base, 3D Printed Robot mount, and EM Probe Mount Locations on the tip of the robot and at the base **(B)** The design of the signal control box showing the input side labeled (1) and output side labeled (2). In addition, the inner workings of the control input and output are shown. The output side is what sends the white light to the base of the sensor and is shown by the heat sink and LED that feeds light into the optical fiber. The input side is what reads the color-shifted response, in which a small mounting system aligns the fiber to the center of the color sensor for accurate readings of RGB values.

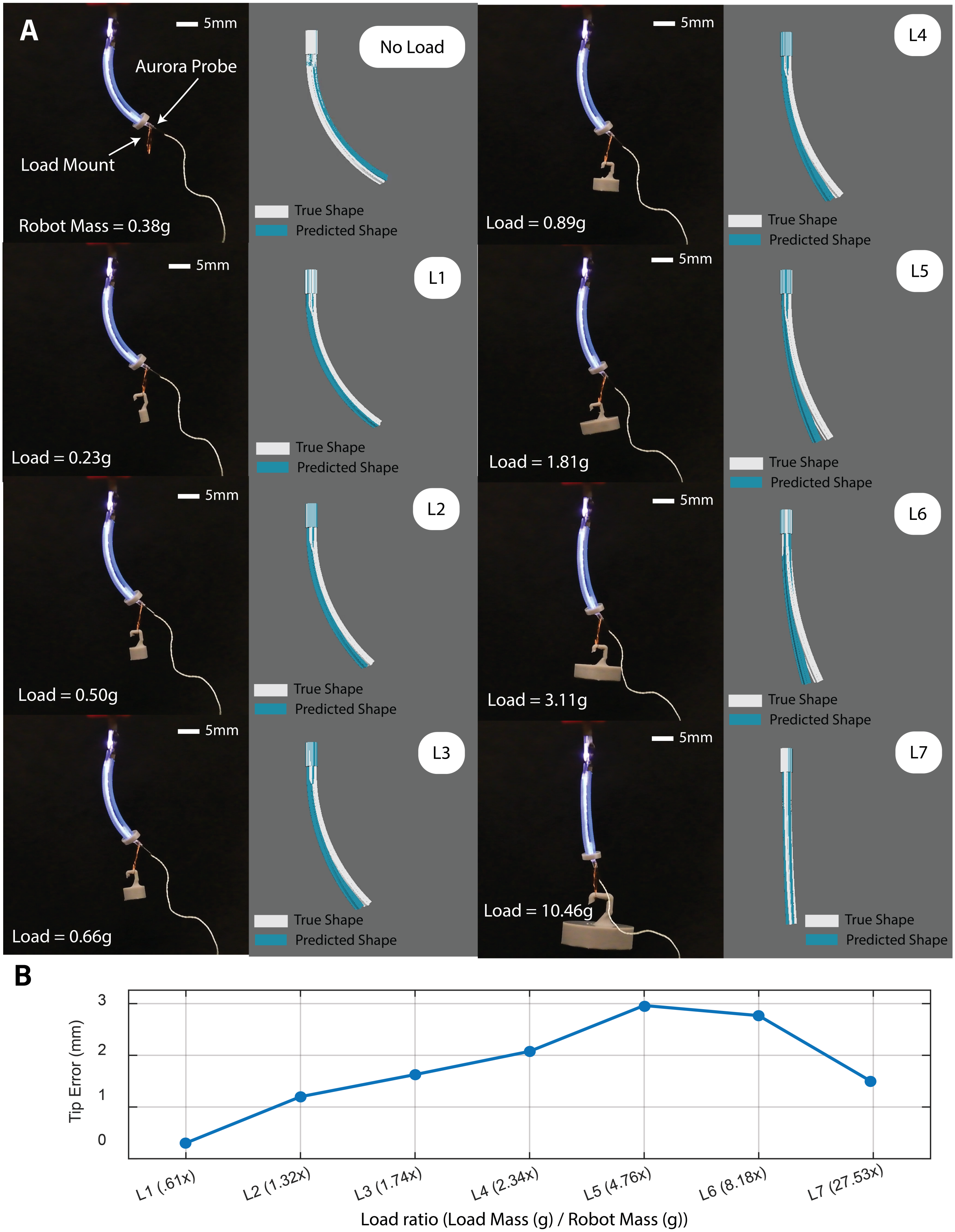

Figure S4 Incremental Load Testing of the Robot and resulting LUMOS Response. **(A)** Pictures for each specific weight are shown, along with the corresponding mass, utilizing a load mount to hold the increasing weight. This is accompanied by the real-time rendered simulation in which the true robot shape (defined by the EM probe) is given in white and the predicted shape of LUMOS is given in blue. **(B)** The tip error for each specific test (L1-L7) is plotted. The load ratio between the robot's mass and the load mass is also given for each test.

Caption for Movie S1. Color Shifting Thin Film Waveguide Core Response: Thin film color-tuned soft core response when manually bent in space, resulting in a visual color-shifting response at the end of the core.

Caption for Movie S2. Manufacturing Overview of LUMOS: The manufacturing process of LUMOS includes the thin film tuning, thin outer WG attachment, and mirror adhesion.

Caption for Movie S3. Optical Color Signal Response as a Result of Bending the Omnidirectional Robot: The color shifting response of LUMOS when embedded into the 3.2 mm soft robotic platform, showing the light color response when each actuator is independently pressurized.

Caption for Movie S4. Real-Time Shape-Sensing Response of the robot as it Moves in 3D Space: The robot's real-time shape sensing response is achieved by processing the resulting RGB color values through the trained neural network. The true vs. predicted real-time 3D rendered output is displayed, in which the white 3D model represents the true shape, while the blue 3D model represents the predicted shape response.

Caption for Movie S5. Closed-Loop Control to Multiple Points in Space: The closed-loop control of the robot to ten points in space in succession is shown. The real-time 3D rendered model in blue is the predicted shape sensing response, while the white model is the target location. The shape sensing response turns green when the target is reached and then moves to the next desired target.

Caption for Movie S6. External Force Control Test: The load response test is shown in which a weight is placed on the actuated robot, and the robot returns to its original position using shape sensing and closed-loop control. The real-time 3D render displays the target location in white, the shape sensing response in blue, which changes to green when the target is reached.

Caption for Movie S7. In-Vitro Autonomous Navigation: The autonomous navigation of the robot with a 3.2 mm diameter through an in-vitro lung environment to two separate target locations is shown. The 3D simulator is displayed alongside the real test, depicting the preoperative path, the robot's shape sensing in blue, and target locations that change to green when reached. After reaching the targets, the robot continues toward the lung exit, as shown by the robot navigating out of the bronchial tree in both the real and simulated videos.
